# Characterization of sound pressure levels and sound sources in the intensive care unit: a 1 week observational study

**DOI:** 10.3389/fmed.2023.1219257

**Published:** 2023-07-13

**Authors:** Aileen C. Naef, Samuel E. J. Knobel, Nicole Ruettgers, Marilyne Rossier, Marie-Madlen Jeitziner, Bjoern Zante, René M. Müri, Joerg C. Schefold, Tobias Nef, Stephan M. Gerber

**Affiliations:** ^1^Gerontechnology and Rehabilitation Group, ARTORG Center for Biomedical Engineering Research, University of Bern, Murtenstrasse, Bern, Switzerland; ^2^Department of Intensive Care Medicine, Inselspital, Bern University Hospital, University of Bern, Freiburgstrasse, Bern, Switzerland; ^3^Institute of Nursing Science (INS), Department of Public Health (DPH), Faculty of Medicine, University of Basel, Bernoullistrasse, Basel, Switzerland; ^4^Department of Neurology, Inselspital, Bern University Hospital, University of Bern, Freiburgstrasse, Bern, Switzerland

**Keywords:** intensive care unit, noise, sound level meters, hospital, decibels, sound pressure levels, sound sources

## Abstract

**Background:**

Exposure to elevated sound pressure levels within the intensive care unit is known to negatively affect patient and staff health. In the past, interventions to address this problem have been unsuccessful as there is no conclusive evidence on the severity of each sound source and their role on the overall sound pressure levels. Therefore, the goal of the study was to perform a continuous 1 week recording to characterize the sound pressure levels and identify negative sound sources in this setting.

**Methods:**

In this prospective, systematic, and quantitative observational study, the sound pressure levels and sound sources were continuously recorded in a mixed medical–surgical intensive care unit over 1 week. Measurements were conducted using four sound level meters and a human observer present in the room noting all sound sources arising from two beds.

**Results:**

The mean 8 h sound pressure level was significantly higher during the day (52.01 ± 1.75 dBA) and evening (50.92 ± 1.66 dBA) shifts than during the night shift (47.57 ± 2.23; *F*(2, 19) = 11.80, *p* < 0.001). No significant difference was found in the maximum and minimum mean 8 h sound pressure levels between the work shifts. However, there was a significant difference between the two beds in the based on location during the day (*F*(3, 28) = 3.91, *p* = 0.0189) and evening (*F*(3, 24) = 5.66, *p* = 0.00445) shifts. Cleaning of the patient area, admission and discharge activities, and renal interventions (e.g., dialysis) contributed the most to the overall sound pressure levels, with staff talking occurring most frequently.

**Conclusion:**

Our study was able to identify that continuous maintenance of the patient area, patient admission and discharge, and renal interventions were responsible for the greatest contribution to the sound pressure levels. Moreover, while staff talking was not found to significantly contribute to the sound pressure levels, it was found to be the most frequently occurring activity which may indirectly influence patient wellbeing. Overall, identifying these sound sources can have a meaningful impact on patients and staff by identifying targets for future interventions, thus leading to a healthier environment.

## 1. Introduction

Prolonged and elevated sound pressure levels in the intensive care unit (ICU) have long been identified as problematic, unduly affecting both patients and healthcare professionals. From a patient perspective, excessive sounds have been found to contribute to negative outcomes, such as cardiovascular stress (1–3), sleep disturbance (4–8), and delirium (5, 9, 10). Similarly, healthcare professionals report negative consequences of high sound pressure levels on their health. Specifically, acute and prolonged exposure has been linked to tachycardia, hypertension, stroke, and burnout (11–14).

Despite these consequences, research has shown that the sound pressure levels in the ICU continuously trend upward (15). Current assessments indicate that the daytime and night-time sound pressure levels in the ICU far exceed the recommended level of 35 dBA and 30 dBA, respectively (12). However, addressing such elevation of levels is challenging, as it often arises directly from patient care involving various medical devices, alarms, and personnel. More specifically, alarms (16, 17), staff behavior (16, 18–20), general activity (19), other patients (8), and equipment (8, 19) all play an essential role in the overall sound pressure levels. Existing studies suggest that over 50% of sound peaks can be attributed to modifiable human behavior, indicating that these sound sources could most easily be addressed through behavioral changes (13, 20, 21). Such sound sources include the ringing of telephones or pagers, talking, and watching of televisions (13, 21). Conversely, non-modifiable sound sources include aspects related to running equipment, such as equipment-related alarms with preset volumes, and life-saving procedures.

However, which of these sound sources occur most frequently and which play the most prominent role in the overall sound pressure level remain inconclusive, with some studies citing staff (16, 20, 22), alarms (23, 24), and daily activities (19, 25). Moreover, comparing or drawing conclusions across studies is challenging owing to the lack of clearly defined methods (25–27). For example, existing studies have performed measurements for anywhere from less than 3 h to 72 h cumulatively, with few studies incorporating any continuous measurement (19–21, 25, 26, 28). Therefore, the understanding of which sound sources are identified and their contribution to the soundscape remains elusive (20, 25). This is problematic when trying to determine which sound sources need to be targeted to decrease the overall sound pressure levels and improve the associated patient and staff outcomes.

Therefore, the goal of this work is to carry out the precise identification of the sound sources and quantify their contribution to the overall sound pressure levels in an ICU setting. Moreover, using the published methods and dataset, future researchers will be able to use this work as a basis for further analyses or as a reference for their results (26). This will ease the implementation of specific interventions to improve the soundscape in the ICU and, consequently, patient and healthcare professionals’ wellbeing (29). Related to this goal, we first hypothesize that an exhaustive list of sound sources from two ICU beds, measured during a continuous 7 day (168 h) period, can be successfully collected. Moreover, we believe that as a result it will be possible to identify roughly for how much time various sound sources occur in this setting. Secondly, we hypothesize that it will be possible to identify which sound sources are responsible for an increase in sound pressure levels greater than 3 dB in the ICU setting (30).

## 2. Materials and methods

### 2.1. Design and study period

This prospective, systematic, and quantitative observational study was conducted between August 23 and August 30, 2021. Follow-up sound pressure levels were recorded from August 30 to September 20, 2021, to ensure that the week during which the observational study was conducted remained unbiased by the presence of the observer. Measurements started and ended at 9:15 a.m. The local ethics committee approved this study via waiver since no identifiable patient data were collected (KEK 2020-01294).

**Figure 1:**
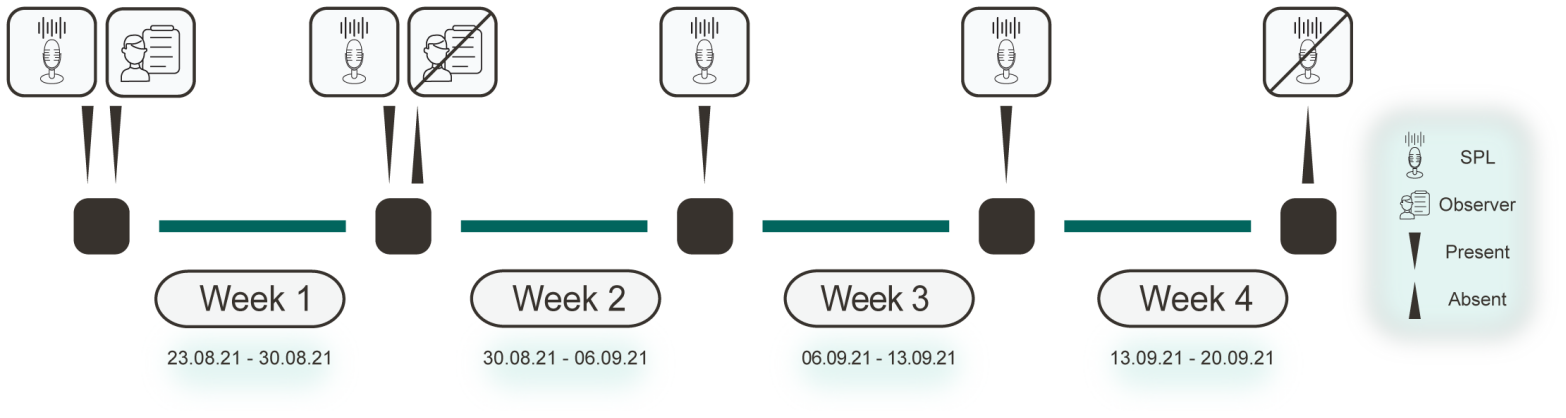
Study procedure. Diagram highlighting when certain aspects were present or absent over the 4  week measurement period. The microphone icon indicates that the SPLs were continuously recorded. The human and paper icon represents the presence of a human observer in the room. SPL, sound pressure level.

Two procedures were conducted during the study: (1) The sound pressure level was measured continuously over 1 month and (2) the sound source was identified continuously for 7 days, concurrent with the first week of the sound pressure level measurements (Figure 1).

### 2.2. Setting

**Figure 2:**
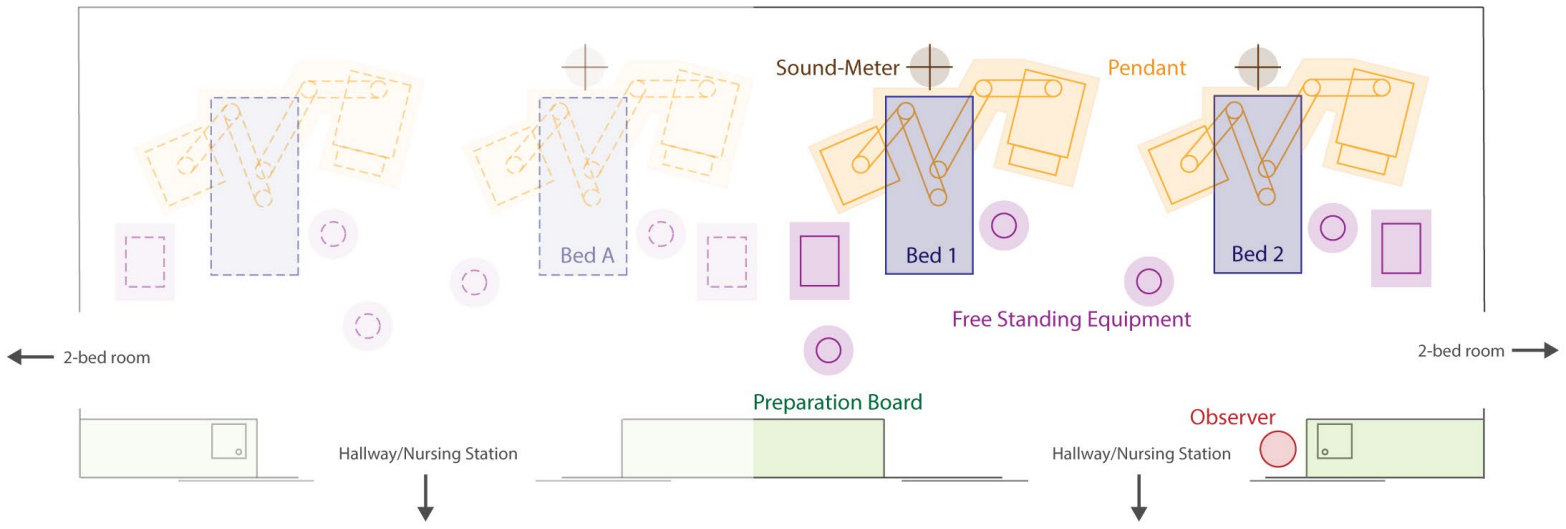
Schematic diagram of ICU rooms. Layout of the four-bed ICU room. The study included two beds (bed 1 and bed 2). Figure adapted from Naef et al. (26). ICU, intensive care unit.

The study was conducted in the mixed medical–surgical ICU at the University Hospital Bern (Inselspital), Switzerland. In this hospital, the ICU comprises three mixed medical–surgical wards, each with 16 beds spread across one-, two-, and four-bed rooms and two central nursing stations. The ward selected for this study had four two-bed and two four-bed rooms. None of the rooms had any sound-proofing materials installed, nor was the ward specifically designed to target elevated sound pressure levels. The study focused on two neighboring beds within a four-bed room [Figure 2 adapted from Naef et al. (23)]. As this study was conducted during the COVID-19 pandemic, a non-COVID-19 ward was selected to ensure that a variety of patients would be present.

### 2.3. Data collection

#### 2.3.1. Sound equipment and measurement

The sound pressure levels were recorded using four sound level meters: one class I device (PCE-430, PCE Germany GmbH, Germany) and three class II devices (Extech-SL400, Extech Instruments, United States; Supplementary Data 1). Class I sound level meters are generally called “precision”-grade devices owing to their wider range. In contrast, class II sound level meters are typically called “general purpose” devices owing to their narrower ranges at higher frequencies (31, 32). The manufacturer calibrated all sound level meters with an accuracy of ±1.4 dB (class II at 1 kHz) and ±1.1 dB (class I at 1 kHz). The class I sound level meter had a sampling rate of 10 Hz, while the class II sound level meters had a sampling frequency of 1 Hz. The measurements were performed using a fast A-frequency weighting method and presented as decibels, as these measurements are comparable to how the human ear responds and are the standard for indoor measurements (31, 33).

The sound level meters were all briefly stopped and restarted every day, so that the data could be collected to minimize the risk of losing data. They were stopped during an observer break, which took 5–10 min on average.

The three class II sound level meters were placed in the four-ICU bed room, one directly above each of the two beds of interest and one above the neighboring bed (Figure 2). The sound level meters were placed approximately 2.5 m above the floor at the head-end of the bed. This was done as the greatest sound pressure levels occur around the head area of the patient bed (22). The class I sound level meter was placed at the central nursing station in the hallway, directly in front of the doors leading to the beds of interest (26). This location was selected based on previously used methods and it’s known relationship with elevated sound pressure levels (13, 17, 22, 34). The doors leading to and between the patient rooms were typically kept open.

#### 2.3.2. Staff number and bed occupancy

Using information provided by the clinic information system of the ICU, we calculated the number of healthcare professionals working in the ICU ward and the bed occupancy (Supplementary Data 1). The healthcare professionals worked in three shifts: day from 7:00 a.m. to 3:00 p.m., evening from 3:00 p.m. to 11:00 p.m., and night from 11:00 p.m. to 7:00 a.m. This also held true on the weekends, except for ICU physicians who worked two 12 h shifts on the weekend: from 8:00 a.m. to 8:00 p.m. and from 8:00 p.m. to 8:00 a.m. Night shifts were classified on the basis of the day they start, not the day they end. Bed occupancy was recorded as one of four possibilities: (1) unoccupied, (2) occupied but absent, (3) occupied and alive, and (4) occupied but deceased. For further analyses, a bed was considered to be unoccupied in the first two cases and occupied in the latter two cases, unless otherwise specified.

### 2.4. Sound source identification

#### 2.4.1. Human observation of sound sources

One human observer was continuously present in the ICU room for 1 week to identify the sources of sounds that occurred. Four observers, whose interrater reliability was tested to ensure no bias was present, rotated through the ICU room (26). All observers had prior experience working in the ICU, either as medical doctors or researchers, and extensively studied and discussed possible sound sources prior to beginning the study. To note the sound sources, the observer used a scoring sheet, which allowed the observer to classify the audible sound sources and note within which 5 min period the sound occurred and for how many minutes. The observer was allowed to take an optional 10 min break per hour between: 20 and: 30 of the hour. Full details and tools used have been previously described by Naef et al. (26). A list of categories of the sound sources that occurred is shown in Supplementary Table S1; Full dataset can be found in Supplementary Data 1.

#### 2.4.2. Devices and alarms

The possibility of retrospectively extracting information from the medical equipment used during the study period was exploited to identify the timing and duration of alarms (Supplementary Data 1). Monitoring alarms, such as alarms for the heart rate, blood pressure, and respiration rate, were all collected using a vital sign monitoring system (Carescape Monitor B650, G.E. Healthcare, United Kingdom) and recorded using the BedMasterEx software (Anandic Medical Systems AG, Switzerland). The monitoring alarms were identified as four types, with a fixed volume: type I, patient advisory alarms (~64 dB); type II, patient warnings (~70 dB); type III, patient crisis alarms (~72.5 dB); and type IV, system alarms (~70 dB). Alarms concerning ventilation were extracted from the ventilator (Hamilton-C6, Hamilton Medical, Switzerland) and categorized into three types according to priority, with no fixed volume. Perfusion alarms (Perfusor Space, B Braun Medical Inc., Germany) also did not have a fixed volume, with warnings and main alarms. Dialysis alarms were extracted from the dialysis devices (Prismaflex, Baxter, United States) and classified as either warnings or main alarms.

Finally, there were miscellaneous alarms such as door alarms or reanimation alarms, which were tracked by the hospital and obtained retrospectively from the clinical information system.

### 2.5. Data analysis

#### 2.5.1. Data preprocessing

After the sound pressure level data exported directly using the manufacturer’s software were collected, they were preprocessed using Python (Python Software Foundation, 2022). Initially, the different days were stitched into a single file. The class I data were then downsampled to 1 Hz, while the class II sound level meters were resampled, so that their timestamps aligned to the full second. The preprocessed sound pressure levels were then used to calculate the equivalent sound pressure level (L_Aeq_). Based on the L_Aeq,_ the minimum (L_AFmin_) and maximum (L_AFmax_) sound pressure levels were calculated per time interval of interest.

After accounting for the breaks taken, we noted that the full duration of the observational data was 148 h and 10 min (8,890 min), instead of 168 h and 0 min (10,080 min). Therefore, to ensure that the data could be accurately compared across work shifts, we resampled the observational data to remove all data recorded during a scheduled break (10 min per hour), regardless of whether it was taken. This ensured that the total duration of each 8 h shift for the analysis, except for the first and last days, was equivalent to 6 h and 40 min (400 min). The duration for 1 day was 20 h and 0 min (1,200 min) or 140 h and 0 min (8,400 min) for 7 days. For data that were not compared directly across work shifts, such as those for the agreement calculation and the linear mixed-effect model, the total dataset was subtracted from the actual breaks taken (148 h and 10 min [8,890 min] for 1 week).

#### 2.5.2. Restorative periods

To quantify the relatively calmer periods for the sound pressure levels, we calculated the restorative periods. The restorative periods were defined as the 5 min periods wherein the L_Aeq,5min_ was <50.0 dBA (35). As the measurements were conducted using a fast time-weighting method, the restorative periods were calculated using the L_Aeq_ only. The number of the restorative periods, mean duration per shift, and five most prolonged restorative periods during week 1 were calculated.

### 2.6. Statistical analysis

All statistical analyses were performed using R statistical language (version 4.2.2; R Core Team, 2022), with the psych (version 2.2.9) and lme4 (version 1.1-31) packages being used for the main analyses.

#### 2.6.1. Sound pressure levels and restorative periods

Descriptive statistics were used to analyze the differences between the 4 week continuous recording data, sound level meter locations, and differences between shifts. Comparisons were made using one-way analysis of variance (ANOVA), with Tukey’s HSD test used for posthoc analyses. One-way ANOVA, with Tukey’s HSD test for posthoc analyses, was also used to investigate the differences between the occurrences and duration of the restorative periods per shift. A dependent two-sample *t*-test was used to analyze the differences between the beds, during each shift, and in the number of occurrences and duration of the restorative periods.

#### 2.6.2. Sound source agreement

To understand whether certain sound sources showed a relationship, we calculated and presented the agreement between the various sound sources using Cohen’s weighted kappa coefficient (*κ*). Only those whose 95% confidence intervals (CIs) were smaller than 1.0 were further examined to determine which results were most relevant.

#### 2.6.3. Effects of the sound sources on the sound pressure levels

To determine which sound sources should be included in the final model, we first modeled each source individually using a linear mixed-effect model. Herein, the goal was two-fold. The first goal was to determine whether the raw data had to be categorized. Specifically, because the preprocessed data were specified as the number of minutes a given sound occurred, an equal distribution between the levels had to be ensured. When the CI of the sound source was greater than 1.50 based on the preprocessed data, the data were grouped into three categories: 0 (no sound present), 1 (sound present for 1 min), or 2 (sound present for ≥2 min). The model was then run again with the new grouping. When the CI was again greater than 1.50, the data were further grouped into two categories: 0 (no sound present) or 1 (sound present). The second goal of the initial model was to determine which of the categorized or non-categorized sound sources should be included in the final model. To do so, we determined that only those whose model estimated a change of ≥3 dB, rounded to the nearest tenth, would be included in the final model. This value was selected, since a healthy ear can perceive only changes in 3 dB (30). Therefore, this was used as a cut-off for the individual sound sources.

The final model included sound sources in their original preprocessed state, with data categorized into three groups or binary data categorized into two groups. The model included the bed (i.e., bed 1 and bed 2) and bed occupancy (1: unoccupied; 2: occupied but absent; 3: occupied and present; and 4: occupied but deceased) as random effects.

## 3. Results

### 3.1. Bed occupancy and staffing

#### 3.1.1. Week 1

During week 1, eight patients (two patients in bed 1 and six patients in bed 2) were treated in the two beds. Bed 1 and bed 2 were unoccupied for 29 h and 35 min and 35 h and 50 min (2,150 min), respectively. This duration included a continuous period of 13 h and 22 min (802 min) wherein both beds were simultaneously unoccupied. Based on a linear regression patient length of stay was not found to have a significant relationship with the overall sound pressure levels (*p* = 0.8879). In the other 14 beds of the ICU ward, 43 additional patients were treated. The overall ward occupancy for week 1 was 74.5% of the 16 beds. The total daily occupancy for the entire ward and the beds of interest is shown in Supplementary Table S4.

**Figure 3:**
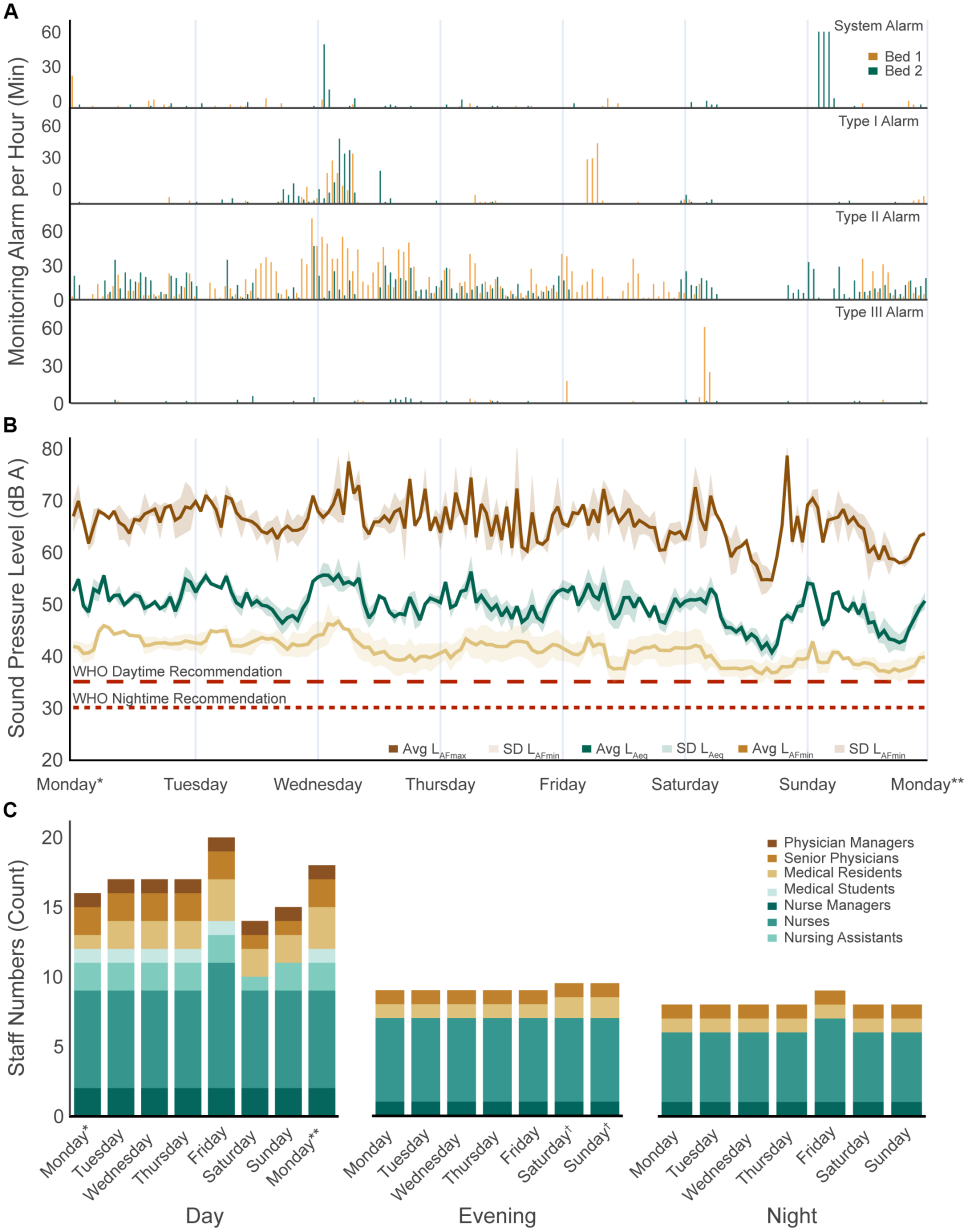
Week 1 sound pressure levels and sound sources. **(A)** Four patient monitoring alarm types present per bed over the 1 week recording period. **(B)** Average equivalent sound pressure level (L_Aeq,1h_) as well as the maximum (L_AFmax,1h_) and minimum (L_AFmin,1h_) levels reached for bed 1 and bed 2 over the 1 week recording period. The dotted lines show the daytime and night-time recommendations by the WHO. **(C)** Number of staff present in the ward per day and shift. WHO, World Health Organization. ^*^Not an entire shift: from study start at 9:15 a.m. to shift end at 03:00 p.m.; ^**^Not an entire shift: from shift start at 7:00 a.m. to study end at 9:15 a.m.; ^†^On weekends, physicians have two instead of three shifts: day shifts start from 8:00 a.m. to 8:00 p.m. and night shifts from 8:00 p.m. to 8:00 a.m. The average of the two values was calculated.

During week 1, there were, on average, 11.12 ± 0.78 nurses and 5.63 ± 1.11 physicians working during the day shift (Figure 3C). During the evening shift, there were, on average, 7.00 ± 0.00 nurses and 2.14 ± 0.23 physicians working (Figure 3C). During the night shift, 6.14 ± 0.35 nurses and 2.00 ± 0.00 physicians on average were working (Figure 3C).

### 3.2. Sound pressure levels

#### 3.2.1. Week 1 to week 4: L_Aeq_, L_AFmax_, and L_AFmin_

In the analysis of the 4 week continuous recordings, there were no significant differences in the L_Aeq,1week_, L_AFmax,1week_, and L_AFmin,1week_ between the weeks for the two beds of interest (Supplementary Table S5).

#### 3.2.2. Week 1: L_Aeq_

The mean L_Aeq,8h_ of the two beds of interest was 52.01 ± 1.75 dBA for the day shift, 50.92 ± 1.66 dBA for the evening shift, and 47.57 ± 2.23 dBA for the night shift (Table 1; Figure 3B). There were significant differences in the L_Aeq,8h_ between the shifts (*F*(2, 19) = 11.80, *p* < 0.001), with the L_Aeq,8h_ during the night shift being significantly lower than that during the day (*p* < 0.001, 95% CI = [−6.84, −2.05]) and evening (*p* = 0.007, 95% CI = [−5.82, −0.88]) shifts.

**Table 1:** Sound pressure levels per shift and day.

Shift		L_Aeq,8h_			
	Day	Hallway (dBA)	Bed 1 (dBA)	Bed 2 (dBA)	Bed A (dBA)
Day	Monday^*^	53.80	52.6	52.0	54.9
	Tuesday	52.55	54.5	53.7	54.3
	Wednesday	53.59	55.7	53.6	54.9
	Thursday	53.68	52.1	50.9	52.7
	Friday	53.72	53.4	51.2	54.2
	Saturday	51.71	51.8	49.3	53.1
	Sunday	52.02	50.8	51.1	52.7
	Monday^**^	52.66	50.8	48.8	52.6
	Mean ± Std	52.97 ± 0.84	52.70 ± 1.76	51.33 ± 1.74	53.67 ± 1.02
Evening	Monday	51.59	52.37	51.73	52.85
	Tuesday	51.06	52.62	50.97	53.34
	Wednesday	50.92	53.36	51.13	53.42
	Thursday	51.47	53.09	49.86	53.50
	Friday	51.95	53.26	49.98	53.57
	Saturday	51.43	48.97	47.17	50.40
	Sunday	50.59	49.97	49.40	51.90
	Mean ± Std	51.29 ± 0.46	51.95 ± 1.75	49.89 ± 1.56	52.71 ± 1.18
Night	Monday	48.31	50.49	49.27	51.17
	Tuesday	49.00	49.32	46.80	51.05
	Wednesday	48.83	49.70	48.57	47.90
	Thursday	47.48	49.38	46.73	50.35
	Friday	47.22	49.77	48.02	49.67
	Saturday	48.39	45.46	43.78	47.55
	Sunday	46.84	45.72	42.94	46.72
	Mean ± Std	48.01 ± 0.83	48.59 ± 2.06	46.59 ± 2.40	49.20 ± 1.80

Summary of the L_Aeq_ per shift (8 h) per day in A-weighted decibels for week 1. The total per shift is calculated as the mean and standard deviation (std) across the days. L_Aeq_, A-weighted time-averaged sound pressure level; hallway, class I sound level meter placed at the nursing station; beds 1, 2, and A, class II sound level meter placed above each bed (26). ^*^Not an entire shift: from study start at 9:15 a.m. to shift end at 3:00 p.m.; ^**^Not an entire shift: from shift start at 7:00 a.m. to study end at 9:15 a.m.

There was a significant difference in the L_Aeq,8h_ according to the sound level meter location during the day (*F*(3, 28) = 3.91, *p* = 0.0189) and evening (*F*(3, 24) = 5.66, *p* = 0.00445) shifts (Table 1). Bed 2 had a significantly lower L_Aeq,8h_ than bed A (bed adjacent to beds of interest) during the day (*p* = 0.0121, 95% CI = [0.423, 4.26]) and evening (*p* = 0.00303, 95% CI = [0.86, 4.78]) shifts. During the evening shift, bed 2 also had a significantly lower (*p* = 0.0375, 95% CI = [0.10, 4.02]) L_Aeq,8h_ than bed 1. During the night shift, there were no significant differences in the L_Aeq,8h_ according to the different sound level meter locations (*F*(3, 24) = 2.49, *p* = 0.0844).

#### 3.2.3. Week 1: L_AFmax_

The mean L_AFmax,8h_ of the two beds of interest was 70.61 ± 3.70 dBA for the day shift, 71.99 ± 3.30 dBA for the evening shift, and 70.56 ± 6.32 dBA for the night shift (Figure 3B; Supplementary Table S2). There were no significant differences in the L_AFmax,8h_ between the shifts (*F*(2, 19) = 0.26, *p* = 0.777).

The maximum level reached differed significantly according to the sound level meter location during both the day (*F*(3, 28) = 9.39, *p* < 0.001) and evening (*F*(3, 24) = 5.70, *p* = 0.00429) shifts (Supplementary Results 1; Supplementary Table S2).

#### 3.2.4. Week 1: L_AFmin_

The mean L_AFmin,8h_ of the two beds of interest was 40.2 ± 1.4 dBA for the day shift, 40.1 ± 2.5 dBA for the evening shift, and 39.5 ± 2.1 dBA for the night shift (Figure 3B; Supplementary Table S3). There were no significant differences in the L_AFmin,8h_ between the shifts (*F*(2, 19) = 0.20, *p* = 0.818).

The minimum level reached differed significantly according to the sound level meter location during the day (*F*(3, 28) = 26.90, *p* < 0.001), evening (*F*(3, 24) = 7.39, *p* < 0.001), and night (*F*(3, 24) = 8.46, *p* < 0.001) shifts (Supplementary Results 1; Supplementary Table S3).

#### 3.2.5. Restorative periods

The total number of the restorative periods (L_Aeq_ < 50 dBA) that occurred during the 1 week observation period when the beds of interest were occupied and unoccupied was 975 (81 h and 15 min [4,875 min]) for bed 1 and 1,351 (112 h and 35 min) for bed 2. The most prolonged restorative period for both beds occurred simultaneously, lasted 12.50 h, and occurred on a weekend while the beds were unoccupied. The details of the five most prolonged restorative periods per bed, not accounting for bed occupancy, are described in Supplementary Results 2 and shown in Supplementary Table S6.

When the restorative periods were considered only when the beds were occupied, bed 1 had 81, 154, and 432 restorative periods for the day, evening, and night shifts, respectively (Table 2). Bed 2 had 223, 251, and 518 restorative periods for the same shifts, respectively (Table 2). There were no significant differences in the mean number of occurrences per day between the shifts for both bed 1 (*F*(2, 16) = 3.12, *p* = 0.0717) and bed 2 (*F*(2, 18) = 3.17, *p* = 0.0662) when occupied. The mean length of the restorative periods significantly differed between bed 1 (1.32 ± 1.08; 0 h and 6 min ± 0 h and 5 min) and bed 2 (3.42 ± 1.74; 0 h and 17 min ± 0 h and 8 min) during the day shift (*t*(13) = −2.75, *p* = .01655) (Table 2).

**Table 2:** Restorative period occurrence and consecutive period.

		Bed occupancy (%)	Max consecutive periods (number of periods)			Occurrences (mean number of periods)	
		Bed 1	Bed 2	Bed 1	Bed 2	Bed 1	Bed 2
Day	Monday^*^	26.09	30.43	0.00	1.00	0.00	1.00
	Tuesday	100.00	97.92	3.00	4.00	1.33	1.67
	Wednesday	100.00	95.83	0.00	6.00	0.00	2.43
	Thursday	100.00	100.00	6.00	11.00	1.80	4.42
	Friday	100.00	54.17	2.00	5.00	1.13	2.71
	Saturday	98.96	100.00	3.00	17.00	2.00	5.92
	Sunday	N/A	64.58	N/A	15.00	N/A	5.20
	Monday^**^	100.00	100.00	9.00	9.00	3.00	4.00
	Mean	89.29 ± 27.87	80.37 ± 27.08	3.29 ± 3.25	8.50 ± 5.55	1.32 ± 1.08	3.42 ± 1.74
Evening	Monday	100.00	95.83	4.00	6.00	1.71	2.24
	Tuesday	100.00	100.00	5.00	10.00	2.13	2.79
	Wednesday	100.00	46.88	10.00	7.00	3.44	3.20
	Thursday	100.00	100.00	11.00	19.00	3.00	6.18
	Friday	61.46	N/A	5.00	N/A	1.83	N/A
	Saturday	N/A	7.29	N/A	0.00	N/A	0.00
	Sunday	31.25	100.00	12.00	30.00	4.75	7.60
	Mean	82.12 ± 29.30	75.00 ± 39.20	7.83 ± 3.54	12.00 ± 10.80	2.81 ± 1.17	3.67 ± 2.77
Night	Monday	100.00	100.00	9.00	16.00	2.84	6.00
	Tuesday	100.00	100.00	25.00	54.00	6.55	30.67
	Wednesday	100.00	100.00	30.00	18.00	8.50	11.14
	Thursday	100.00	100.00	25.00	49.00	8.44	18.20
	Friday	100.00	76.04	31.00	31.00	7.44	10.17
	Saturday	N/A	26.04	N/A	18.00	N/A	5.50
	Sunday	100.00	100.00	94.00	96.00	47.50	96.00
	Mean	100.00 ± 0.00	86.01 ± 27.91	35.70 ± 29.60	40.30 ± 29.00	13.50 ± 16.80	25.40 ± 32.30

N/A, not applicable (bed unoccupied for the entire shift); ^*^Not an entire shift: from study start at 9:15 a.m. to shift end at 3:00 p.m.; ^**^Not an entire shift: from shift start at 7:00 a.m. to study end at 9:15 a.m.

The maximum number of consecutive periods was significantly different between the shifts for bed 1 (*F*(2, 16) = 5.46, *p* = .0134) and bed 2 (*F*(2, 18) = 7.31, *p* = .00442; Table 2). The posthoc test revealed that the maximum average number of consecutive periods reached was significantly larger during the night shift than during the day (bed 1: *p* = .01563, 95% CI = [4.98, 50.42]; bed 2: *p* = .00693, 95% CI = [8.50, 55.07]) and evening (bed 1: p = .04591, 95% CI = [0.39, 47.32]; bed 2: *p* = .01336, 95% CI = [5.95, 54.05]) shifts for both beds (Table 2).

### 3.3. Sound sources

#### 3.3.1. Objective sound sources: alarms generated

During week 1, the equipment generated alarms for 94 h and 5 min (5,645 min). Specifically, patient monitoring alarms occurred for 64 h and 44 min (3,884 min) (Figure 3A), ventilator alarms for 22 h and 58 min (1,378 min), door alarms for 4 h and 2 min (242 min), perfusion alarms for 2 h and 9 min (129 min), and dialysis alarms for 0 h and 12 min (12 min) across the two beds (Table 3).

**Table 3:** Duration of occurrence of different human and object sound sources over week 1.

	Description	Bed 1				Bed 2			
		Overall	Day	Evening	Night	Overall	Day	Evening	Night
Human (–Human) Sounds	Staff < 3 talking (out-of-ward rounds)	2,248 min	25.71%	26.43%	28.14%	1,536 min	27.46%	17.96%	9.43%
	Staff ≥ 3 talking (out-of-ward rounds)	370 min	4.79%	4.96%	3.46%	346 min	6.64%	3.68%	2.04%
	Staff talking with the patient	833 min	8.82%	11.00%	9.93%	948 min	15.39%	8.86%	9.61%
	Visitors, staff, and patient talking ≥ 3 people	359 min	4.75%	8.07%	0.00%	14 min	0.00%	0.04%	0.46%
	Patient sounds	176 min	3.25%	0.64%	2.39%	445 min	6.96%	3.25%	5.68%
Object (–Human Interaction) Sounds	Ventilatory interventions	1,263 min	8.07%	19.39%	17.64%	365 min	0.57%	6.75%	5.71%
	Cardiovascular interventions	6 min	0.00%	0.00%	0.21%	2064 min	12.07%	38.29%	23.36%
	Preparation board	1780 min	19.89%	21.82%	21.86%	539 min	10.75%	5.54%	2.96%
	Free standing equipment	549 min	5.14%	7.11%	7.36%	604 min	9.75%	5.14%	6.68%
	Medical Pendant	1,157 min	14.54%	15.75%	11.04%	1,066 min	15.71%	13.04%	9.32%
	Clothing accessories	845 min	10.25%	12.93%	7.00%	561 min	9.68%	6.61%	3.75%
Monitoring Alarms	Patient Advisory	306 min	5.39%	5.14%	0.39%	268 min	4.82%	3.50%	1.25%
	Patient Warning	1,697 min	21.71%	20.32%	18.57%	1,137 min	16.64%	12.50%	11.46%
	Patient Crisis	96 min	2.43%	0.32%	0.04%	34 min	0.32%	0.35%	0.54%
	System	76 min	0.29%	0.64%	1.07%	270 min	8.75%	0.64%	0.25%
Ventilation Alarms	Type I	38 min	0.50%	0.39%	0.46%	28 min	0.36%	0.29%	0.36%
	Type II	797 min	13.71	6.82%	7.93%	124 min	0.68%	2.54%	1.21%
	Type III	305 min	4.82	3.11%	2.956	86 min	0.36%	1.32%	1.39%

Overall time of the occurrence of the sound sources in minutes over the 1 week observation period (8,400 min excluding the breaks) and percentage of occurrence per 8 h shift (minus the breaks, 2,800 min). The top five human (–human) sounds and the top six object (–human) interaction sounds (as determined by the total sum between the two beds) in addition to the patient monitoring alarms and ventilation equipment alarms are shown. The results for each category are in Supplementary Table S5.

#### 3.3.2. Subjective sound sources: human (–human) and object (–human interaction) generated

Considering the observer breaks during week 1 (140 h; 8,400 min), human (–human interaction) sounds occurred for 69 h and 21 min (4,161 min, 49.54%) for bed 1 and 58 h and 12 min (3,492 min, 41.57%) for bed 2. The greatest contributor to the overall duration of human (–human interaction) sounds was one to two staff members talking outside of ward rounds, which occurred for 37 h and 28 min (2,248 min, 26.76%) for bed 1 and 25 h and 36 min (1,536 min, 18.29%) for bed 2 (Table 3). Over week 1, object (–human interaction) sounds, not including alarms, occurred for 106 h and 58 min (6,418 min, 76.41%) for bed 1 and 108 h and 36 min (6,516 min, 77.57%) for bed 2. For bed 1, the sources responsible for the highest percentage of sounds were preparation boards (21.19%, 29 h and 40 min, 1780 min), ventilatory interventions (15.04%, 21 h and 3 min, 1,263 min), medical pendants (13.77%, 19 h and 17 min, 1,157 min), clothing accessories (10.06%, 14 h and 5 min, 845 min), and free-standing equipment (6.54%, 9 h and 9 min, 549 min). For bed 2, cardiovascular interventions were the greatest contributor of the sounds (24.57%, 34 h and 24 min, 2064 min), followed by medical pendants (12.69%, 17 h and 46 min, 1,066 min), free-standing equipment (7.19%, 10 h and 4 min, 604 min), clothing accessories (6.68%, 9 h and 21 min, 561 min), preparation boards (6.42%, 8 h and 59 min, 539 min), and ventilatory interventions (4.35%, 6 h and 5 min, 365 min). The five human (–human interaction) sounds and the six object (–human interaction) sounds that occurred for the greatest amount of time are presented in Table 3, with the full results presented in Supplementary Tables S7–S15.

#### 3.3.3. Sound source agreement

In the analysis of the agreement between the sound sources whose CI was smaller than 1, the highest agreement was noted between type II monitoring alarms and one to two staff members talking outside of ward rounds (*κ* = 0.28, CI = [0.11, 0.44]; Table 4). The subsequent five highest agreements were found between one to two staff members talking outside of ward rounds and medical pendants (*κ* = 0.27, CI = [0.05, 0.48]), preparation boards (*κ* = 0.21, CI = [−0.02, 0.45]), and staff talking with patients (*κ* = 0.19, CI = [−0.11, 0.48]) as well as between type II monitoring alarms and medical pendants (*κ* = 0.24, CI = [−0.06, 0.53]) and ventilatory interventions (*κ* = 0.19, CI = [−0.18, 0.56]; Table 4).

**Table 4:** Agreement between the sound sources.

Variable 1	Variable 2	Weighted *κ*	Lower Conf. Int (95%)	Upper Conf. Int (95%)	The difference in Conf. Int
Monitoring Alarm Type II	SToWR12	0.28	0.11	0.44	0.33
SToWR12	Medical Pendant	0.27	0.05	0.48	0.44
SToWR12	Preparation Board	0.21	−0.02	0.45	0.46
SToWR12	Staff Talking with Patient	0.19	−0.11	0.48	0.59
Monitoring Alarm Type II	Medical Pendant	0.24	−0.06	0.53	0.59
Ventilator Alarm Type II	SToWR12	0.13	−0.17	0.44	0.6
SToWR12	Ventilatory Int.	0.13	−0.18	0.45	0.63
SToWR12	Cardiovascular Int.	0.00	−0.34	0.35	0.69
SToWR12	Clothing Accessories	0.13	−0.22	0.47	0.69
Monitoring Alarm Type II	Preparation Board	0.10	−0.26	0.46	0.71
SToWR12	Free Standing Equipment	0.15	−0.2	0.50	0.71
Monitoring Alarm Type I	SToWR12	0.02	−0.35	0.38	0.73
Hamilton Alarm Type III	SToWR12	0.06	−0.3	0.43	0.73
Monitoring Alarm Type II	Ventilatory Int.	0.19	−0.18	0.56	0.74
Monitoring Alarm System	SToWR12	0.00	−0.39	0.38	0.78
Door Alarm	SToWR12	0.01	−0.38	0.4	0.78
Monitoring Alarm Type III	SToWR12	0.01	−0.39	0.41	0.80
Monitoring Alarm Type II	Cardiovascular Int.	0.05	−0.35	0.45	0.80
SToWR12	SToWR3	0.14	−0.25	0.54	0.80
Perfusion Alarm	SToWR12	0.01	−0.40	0.42	0.81
Ventilator Alarm Type I	SToWR12	0.01	−0.39	0.42	0.81
Cardiovascular Int.	Medical Pendant	0.05	−0.35	0.46	0.81
STWR12	SToWR12	0.00	−0.41	0.41	0.82
Perfusion Pre Alarm	SToWR12	0.00	−0.41	0.41	0.82
Ventilatory Int.	Preparation Board	0.12	−0.29	0.53	0.82
Ventilator Alarm Type II	Monitoring Alarm Type II	0.18	−0.25	0.62	0.87
SToWR12	Bed	0.10	−0.37	0.56	0.92
Cardiovascular Int.	Preparation Board	−0.10	−0.57	0.37	0.93
Staff Talking with Patient	Medical Pendant	0.20	−0.27	0.67	0.94
Ventilatory Int.	Medical Pendant	0.05	−0.43	0.53	0.96
Monitoring Alarm Type II	Free Standing Equipment	0.15	−0.33	0.63	0.96
Monitoring Alarm Type II	Clothing Accessories	0.09	−0.40	0.57	0.97
Preparation Board	Medical Pendant	0.15	−0.34	0.64	0.98

Weighted *κ*, weighted kappa; lower conf. Int. (95%), lower limit of the 95% confidence interval; upper conf. Int. (95%), upper limit of the 95% confidence interval; difference in conf. Int., difference between the upper and lower 95% confidence interval; SToWR12, staff talking outside of ward rounds (1–2 people); SToWR3, staff talking outside of ward rounds (>3 people); STWR12, staff talking during ward rounds (1–2 people); int., intervention.

### 3.4. Effects of the sound sources on the sound pressure levels

Using the linear mixed-effect model, we evaluated the effects of the various sound sources on the sound pressure level of bed 1 and bed 2 (formula: L_Aeq_ ~ sound sources). The total explanatory power of the random-and fixed-effect model was *R*^2^ of 0.44 (conditional *R*^2^), with fixed effects accounting for 0.34 (marginal *R*^2^) of the variance alone. The intercept of the model was at 47.16 (95% CI = [45.46, 48.85], *p* < 0.001). Numerous sound sources were found to show a significant increase within the model (Table 5). Those presenting a change of greater than 3 dB were renal interventions (estimate = 3.87, 95% CI = [3.14, 4.60], *p* < 0.001) and continuous maintenance (estimate = 3.98, 95% CI = [3.37, 4.58], *p* < 0.001; Table 5). The five next highest estimates were noted with admission and discharge (estimate = 2.97, 95% CI = [2.00, 3.94], *p* < 0.001), three or more staff members talking outside of ward rounds for ≥2 min (estimate = 2.62, 95% CI = [2.14, 3.10], *p* < 0.001), radiological intervention (estimate = 2.27, 95% CI = [1.35, 3.19], *p* < 0.001), type II monitoring alarm for 5 min (estimate = 2.26, 95% CI = [1.80, 2.72], *p* < 0.001), and nursing tasks (estimate = 3.87, 95% CI = [1.39, 2.75], *p* < 0.001; Table 5). An increased duration of the sound sources increased the model estimates {e.g., type II monitoring alarm with a duration of 2 min [estimate = 0.87, 95% CI = (0.44, 1.30), *p* < 0.001] versus a duration of 5 min [estimate = 2.26, 95% CI = (1.80, 2.72), *p* < 0.001]; Table 5}.

**Table 5:** Linear mixed-effect model: L_Aeq_ 5 min.

Fixed effects	L_Aeq_		
Coefficient	Estimates	Conf. Int (95%)	*p*-value
Intercept	47.16	[45.46, 48.85]	**<0.001**
Monitoring Alarm Type II (1 min)	0.35	[−0.02, 0.71]	0.063
Monitoring Alarm Type II (2 min)	0.87	[0.44, 1.30]	**<0.001**
Monitoring Alarm Type II (3 min)	0.81	[0.35, 1.26]	**0.001**
Monitoring Alarm Type II (4 min)	1.29	[0.75, 1.83]	**<0.001**
Monitoring Alarm Type II (5 min)	2.26	[1.80, 2.72]	**<0.001**
Ventilator Alarm Type II (1 min)	0.47	[0.01, 0.93]	**0.046**
Ventilator Alarm Type II (≥2 min)	0.95	[0.48, 1.42]	**<0.001**
Ventilator Alarm Type III (1 min)	1.23	[0.66, 1.80]	**<0.001**
Ventilator Alarm Type III (≥2 min)	1.57	[0.87, 2.27]	**<0.001**
Staff 1–2 people talking (out of ward rounds) (1 min)	1.03	[0.74, 1.32]	**<0.001**
Staff 1–2 people talking (out of ward rounds) (≥2 min)	1.73	[1.47, 1.99]	**<0.001**
Staff >3 people talking (out of ward rounds) (1 min)	1.06	[0.54, 1.59]	**<0.001**
Staff >3 people talking (out of ward rounds) (≥2 min)	2.62	[2.14, 3.10]	**<0.001**
Staff Talking with Patient (1 min)	0.02	[−0.35, 0.39]	0.925
Staff Talking with Patient (≥2 min)	1.12	[0.78, 1.46]	**<0.001**
Ventilator Alarm Type I	0.14	[−0.83, 1.11]	0.778
Admission/Discharge	2.97	[2.00, 3.94]	**<0.001**
Activities of Daily Living	1.22	[0.53, 1.91]	**0.001**
Nursing	2.07	[1.39, 2.75]	**<0.001**
Radiological Intervention	2.27	[1.35, 3.19]	**<0.001**
Neurological Intervention	1.55	[−0.37, 3.48]	0.114
Renal Intervention	3.87	[3.14, 4.60]	**<0.001**
Unknown Intervention	1.24	[−0.31, 2.80]	0.117
Bed	1.19	[0.79, 1.59]	**<0.001**
Continuous Maintenance	3.98	[3.37, 4.58]	**<0.001**
Random Effects			
*σ* ^2^	9.16		
ICC	0.15		
τ_00Bed_/τ_00Occupancy_	1.24/0.41		
*N*_Bed_/*N*_Occupancy:_	2/4		
Observations	3,556		
Marginal *R*^2^/Conditional *R*^2^	0.341/0.441		

Output from the linear mixed-effect model, with the dependent variable being the A-weighted time-averaged sound pressure level (L_Aeq_) over 5 min intervals for the two beds of interest. Observations, number of samples in the model; marginal *R*^2^, proportion of variance explained by the fixed factors; conditional *R*^2^, proportion of variance explained by the random factors; conf. Int. (95%), 95% confidence interval. Bold values in Table 5 indicate those that are significant (*p* < 0.05).

## 4. Discussion

Within our 1 week continuous observation period, and in line with the goal of this work, we were able to identify the characteristics of the sound pressure levels across work shifts and sound level meter locations in the ICU. Specifically, we were able to confirm our first hypothesis by showing that it is possible to successfully identify and collect an exhaustive list of sound sources, and their duration of occurrence, over a continuous 7 day period. Furthermore, in line with our second hypothesis, it was possible to identify the greatest contributors to the overall sound pressure levels in our setting. This is an important step for the future implementation of effective interventions aimed at lowering these levels. Moreover, our work, based on a previously validated and repeatable method, acts as a reference for future work on this topic (26).

The results of our work showed no differences in the overall sound pressure levels, nor the maximum and minimum levels reached, during the 4 week recording period. Therefore, while it is possible that the individual sound sources differed from 1 week to another, these differences did not impact the overall sound pressure levels. These findings also highlight that even if some bias was introduced by the presence of an observer in the room, such bias was not sufficient to alter the overall sound pressure levels during the observation week. This notion was further examined in prior work and limited here in a few ways (26). Firstly, the staff were informed about the study and instructed not to interact with the study team members and to continue working normally. Additionally, the staff were informed that no identifying information was being written down, nor was the content of their conversations being noted. Finally, the continuous presence of an observer in the room makes it unlikely that staff would have been able to maintain any altered behavior for a prolonged period, should they have made adaptations.

Consistent with the literature, the L_Aeq_ recorded during this study confirms that the overall decibel levels surpass both the World Health Organization’s recommendations (35 dBA daytime; 30 dBA night time) and that of the Environmental Protection Agency (45 dBA daytime; 35 dBA nighttime) (12, 36). This is in line with findings from Busch-Vischniac et al. showing that no previously published results have found a hospital that complies with these recommendations (15). Moreover, the results found here, while above recommended levels, were lower than findings from other studies (16, 17, 19). Moreover, the minimum levels reached approximately 40 dBA levels during each shift, which is below the daytime recommendation set forth by the Environmental Protection Agency (36).

Regarding the restorative periods (L_Aeq_ < 50 dBA), the results highlight fewer restorative periods during the day shift than during the evening and night shifts. Furthermore, the restorative periods were most noted during the night shift but more frequently occurred on weekends than on weekdays. This finding is in line with the literature and what is expected, as there are fewer staff working and fewer interventions scheduled on weekends (17). The restorative periods in our study, particularly at night, are longer than those in the study by Ryherd et al. (35).

Apart from having significantly more restorative periods than bed 1, bed 2 was also significantly quieter than bed 1 and bed A. When the position of bed 2 was considered, it could be seen that this bed was bordered on two sides by walls. However, Darbyshire et al. (22) found that the majority of sounds in a four-ICU bed room came from areas adjacent to the head of patients, suggesting that sounds do not come from the sides of beds or neighboring beds. This latter notion is supported by the report by Tegnestedt et al. (17), who found no significant difference in the mean sound pressure levels between room types (i.e., one-versus three-bed rooms). However, this study found that disruptive sounds occurred less frequently in one-bed rooms than in three-bed rooms. Considering the layout of the four-bed room where the present study was conducted, it seems feasible that the beds in the middle of the room were louder owing to the presence of a preparation board at the foot end of these beds (Figure 2). This preparation board is the main area in the room where nurses for all four beds prepare medication, gather supplies, and often hold conversations. The results from our agreement calculation support this notion, showing a trend in the agreement between one to two staff members talking outside of ward rounds and activity at the preparation board. The preparation board was also found to display trends in agreement with type II patient monitoring alarms, cardiovascular interventions, ventilatory interventions, and medical pendants, suggesting that activity at the preparation board is often related to time-sensitive actions. However, this does not rule out that the elevated sound pressure levels recorded at bed A arise due to specific patient care taking place there.

These activities were also among the top sound sources, further supporting the notion that these activities may overlap in occurrence. Moreover, they largely align with the main sound sources found in the literature (16, 17, 20). Among the other top sound sources were staff talking among each other, with patients, and with visitors. Notably, staff talking during ward rounds, wherein multiple individuals enter the patient room simultaneously, was not considered an important contributor to the overall sound pressure levels. However, this may be attributed to the short duration of occurrence. Therefore, while moving ward rounds to a quieter location may be beneficial for information processing, it may have a limited influence on decreasing the sound pressure levels in the room (37). Another explanation as to why talking between individuals was not a significant contributor to the overall sound pressure levels could be explained by the mask requirement during this period. As all individuals entering the ICU were required to wear a non-fabric surgical mask this could have attenuated the sound pressure levels generated (38). As such, this is a limitation of our work but remains relevant in ICUs that still enforce mask mandates. Irrespective of their role on the overall sound pressure levels, talking activities may still affect patients. This is because humans typically perceive sounds with comprehensible content, such as speech, as more disturbing than those without, as well as sudden and short-lasting sounds as more bothersome than continuous sounds (39, 40). This also touches on the fact that how patients and staff perceive a sound can influence its tolerability. This is important because while previous work examining this question in healthcare professionals has found that they perceived short-lasting human sounds (e.g., shouts, laughing, moaning) as most disturbing, this was not found to be a significant contributor to the overall sound pressure levels in our study. Therefore, it would be important for future work to examine how the greatest contributors to sound pressure levels, and the sound sources that occur most often, are perceived by patients and staff.

That being said, the results of the linear mixed-effect model point to two main activities that could be targeted to decrease the overall sound pressure levels in the room. Specifically, continuous maintenance in the room, including emptying, cleaning, and preparation of the room for another patient, was responsible for an almost 4 dB increase in the overall sound pressure level, with the arrival or discharge of a patient accounting for almost an additional 3 dB increase. Therefore, activities related to these procedures should be further investigated to decrease the overall sound pressure levels in the ICU. Renal interventions, such as dialysis, also accounted for an almost 4 dB increase in the overall sound pressure levels. This is likely attributed to the continuous sound produced by the dialysis machine, rather than any dialysis-related alarms, which may imply that such interventions should potentially be moved into designated areas to reduce the overall sound pressure level in shared rooms. Our results also suggest that addressing alarms as rapidly as possible can also help decrease the overall sound pressure levels. While our results are in line with those by Vreman et al. (33), who also concluded that alarms do not greatly contribute to the mean sound pressure levels in the ICU, we found that the amount alarms contribute increases in line with their duration. Therefore, we do not exclude the possibility that alarms can become significant contributors to the overall sound pressure levels should they be allowed to continue for prolonged periods.

Accordingly, we cannot conclude that reducing medical equipment alarms is the best approach for reducing the sound pressure levels, as expressed as desirable by healthcare professionals (40). Instead, our results clearly demonstrate that specific activities and processes should be targeted to decrease the overall sound pressure level. We specifically recommend that future studies investigate how processes such as cleaning and preparing patient rooms and admitting and discharging patients are conducted and how they may be adapted to decrease their contribution to the sound pressure levels. Moreover, interventions such as renal, radiological, cardiovascular, and ventilatory interventions may be further studied to identify why these interventions greatly contribute to the overall levels. The findings may be used to study how aspects of such interventions could be transferred to locations further from patient beds. However, the feasibility of making such changes will remain challenging owing to the structural limitations of ICU wards and patient rooms. A more feasible change may be to address clothing accessories. Perhaps a less expected sound source, clothing accessories were among the most frequently noted sound sources occurring approximately 10% of the time in bed 1 and 7% in bed 2. These sounds arise from objects attached to the clothing, such as keys, badges, and pens, hitting each other while walking or even hitting the bed or equipment while bending over. Although seemingly insignificant, targeting such aspects of the ICU environment could be a simple and efficient way of reducing a recurrent sound source.

### 4.1. Limitations

An important limitation of this study is that not all sound sources occurred equally, which must be considered when comparing them. Nevertheless, the week selected for the study was likely representative of an average week in terms of the sound pressure levels and representative of real working conditions. Future work could consider this and investigate how the various sound sources compare when corrected for their frequency of occurrence. Due to a second limitation, owing to the study design, we were unable to determine whether certain diagnoses were associated with higher sound pressure levels than others, but which could be explored in future work. Additionally, owing to the sheer number of sounds produced, it was impossible to note what aspects of a sound source were responsible for the greatest contribution to the sound pressure levels. For example, during the various interventions, it was impossible to determine whether the sound came from the opening of the packaging, the equipment used, or some other source responsible for contributing the most to the sound pressure levels. These aspects of sound sources could interest future researchers and enable the creation of interventions for decreasing the sound pressure levels in a given setting.

Additional limitations faced by our study include the lack of sleep assessment, meaning that it was not possible to determine how the sound pressure levels and sound sources highlighted in this study affect patient sleep. This would be important to investigate in future research due to the relationship between sleep disruption and various psychophysiological effects, which can in turn increase pain perception, hinder recovery, and increase mortality (41–43). Moreover, our study failed to conduct an objective measurement of the baseline background sound pressure levels created by structural systems, for example any air filtration systems. During the measurement period the ward was always in use and the room occupied by at minimum one patient. Therefore, nothing can be said about how such air filtration systems contribute to the overall sound pressure levels, or whether other sounds are louder because of this (e.g., talking louder to overcome the baseline).

## 5. Conclusion

Decreasing the elevated sound pressure levels that continuously surpass recommended levels is an important step to improve patient and staff wellbeing in the intensive care unit. Findings from our 1 week continuous recording have found that to achieve this, future interventions should target continuous maintenance of the patient area, patient admissions and discharges, and renal interventions; these sound sources are responsible for the greatest changes in the sound pressure levels and are detectable by the human ear. We also determined that one to two staff members talking in the room occurred for the greatest amount of time, and while not a significant contributor to the overall sound pressure levels, may still play an indirect role in patient wellbeing. Overall, identifying these sound sources can have a meaningful impact on patients and staff by identifying targets for future interventions to decrease the sound pressure levels, thus leading to a healthier environment.

## Data availability statement

The original contributions presented in the study are included in the article/Supplementary material, further inquiries can be directed to the corresponding author.

## Author contributions

AN, NR, SK, and SG designed the study, collected data, analyzed the data, and were involved in the writing, editing, and reviewing of the manuscript. MR, M-MJ, BZ, RM, JS, and TN assisted in the data collection and study design, and were involved in editing and reviewing the manuscript. All authors contributed to the article and approved the submitted version.

## Funding

Open access funding by University Of Bern.

## Conflict of interest

M-MJ, BZ, and JS report grants from Orion Pharma, Abbott Nutrition International, B. Braun Medical AG, CSEM AG, Edwards Lifesciences Services GmbH, Kenta Biotech Ltd., Maquet Critical Care AB, Omnicare Clinical Research AG, Nestle, Pierre Fabre Pharma AG, Pfizer, Bard Medica S.A., Abbott AG, Anandic Medical Systems, Pan Gas AG Healthcare, Bracco, Hamilton Medical AG, Fresenius Kabi, Getinge Group Maquet AG, Dräger AG, Teleflex Medical GmbH, Glaxo Smith Kline, Merck Sharp and Dohme AG, Eli Lilly, and Company, Baxter, Astellas, Astra Zeneca, CSL Behring, Novartis, Covidien, Hemotune, Phagenesis, Philips Medical, Prolong Pharmaceuticals, and Nycomed outside the submitted work. The money received was paid into departmental funds. No personal financial gain was applied.

The remaining authors declare that the research was conducted in the absence of any commercial or financial relationships that could be construed as a potential conflict of interest.

## Publisher’s note

All claims expressed in this article are solely those of the authors and do not necessarily represent those of their affiliated organizations, or those of the publisher, the editors and the reviewers. Any product that may be evaluated in this article, or claim that may be made by its manufacturer, is not guaranteed or endorsed by the publisher.

## References

[1] Aaron JN Carlisle CC Carskadon MA Meyer TJ Hill NS Millman RP. Environmental noise as a cause of sleep disruption in an intermediate respiratory care unit. Sleep. (1996) 19:707–10. doi: 10.1093/sleep/19.9.707, PMID: 9122557

[2] Basner M Babisch W Davis A Brink M Clark C Janssen S. Auditory and non-auditory effects of noise on health. Lancet. (2014) 383:1325–32. doi: 10.1016/S0140-6736(13)61613-X 24183105 PMC3988259

[3] Morrison WE Haas EC Shaffner DH Garrett ES Fackler JC. Noise, stress, and annoyance in a pediatric intensive care unit. Crit Care Med. (2003) 31:113–9. doi: 10.1097/00003246-200301000-00018, PMID: 12545003

[4] Freedman NS Gazendam J Levan L Pack AI Schwab RJ. Abnormal sleep/wake cycles and the effect of environmental noise on sleep disruption in the intensive care unit. Am J Respir Crit Care Med. (2001) 163:451–7. doi: 10.1164/ajrccm.163.2.9912128, PMID: 11179121

[5] Honarmand K Rafay H Le J Mohan S Rochwerg B Devlin JW. A systematic review of risk factors for sleep disruption in critically ill adults. Crit Care Med. (2020) 48:1066–74. doi: 10.1097/CCM.0000000000004405, PMID: 32433122

[6] Kamdar BB Needham DM Collop NA. Sleep deprivation in critical illness: its role in physical and psychological recovery. J Intensive Care Med. (2012) 27:97–111. doi: 10.1177/0885066610394322, PMID: 21220271 PMC3299928

[7] Topf M Bookman M Arand D. Effects of critical care unit noise on the subjective quality of sleep. J Adv Nurs. (1996) 24:545–51. doi: 10.1046/j.1365-2648.1996.22315.x, PMID: 8876415

[8] Delaney LJ Currie MJ Huang H-CC Lopez V Van Haren F. “They can rest at home”: an observational study of patients’ quality of sleep in an Australian hospital. BMC Health Serv Res. (2018) 18:524. doi: 10.1186/s12913-018-3201-z, PMID: 29976191 PMC6034217

[9] Sangari A Emhardt EA Salas B Avery A Freundlich RE Fabbri D. Delirium variability is influenced by the sound environment (DEVISE study): how changes in the intensive care unit soundscape affect delirium incidence. J Med Syst. (2021) 45:76. doi: 10.1007/s10916-021-01752-5, PMID: 34173052 PMC8300597

[10] van de Pol I van Iterson M Maaskant J. Effect of nocturnal sound reduction on the incidence of delirium in intensive care unit patients: an interrupted time series analysis. Intensive Crit Care Nurs. (2017) 41:18–25. doi: 10.1016/j.iccn.2017.01.008, PMID: 28351551

[11] Alidosti M Babaei Heydarabadi A Baboli Z Nazarbigi H Mobasheri M. Association between job burnout and noise pollution among nurses in Behbahan city. Iran Q J Fundam Mental Health. (2016) 18:103–8. doi: 10.22038/JFMH.2016.6676

[12] Berglund B Lindvall T Schwela DH Organization WH. Guidelines for community noise. Stockholm Sweden: World Health Organization (1999). 1999 p.

[13] Pugh RJ Jones C Griffiths R. Editors. The impact of noise in the intensive care unit. Intensive care medicine: annual update 2007 Springer (2007).

[14] Topf M Dillon E. Noise-induced stress as a predictor of burnout in critical care nurses. Heart Lung. (1988) 17:567–74. PMID: 3417467

[15] Busch-Vishniac IJ West JE Barnhill C Hunter T Orellana D Chivukula R. Noise levels in Johns Hopkins Hospital. J Acoust Soc Am. (2005) 118:3629–45. doi: 10.1121/1.2118327, PMID: 16419808

[16] Delaney LJ Currie MJ Huang HC Lopez V Litton E Van Haren F. The nocturnal acoustical intensity of the intensive care environment: an observational study. J Intensive Care. (2017) 5:41. doi: 10.1186/s40560-017-0237-9, PMID: 28702196 PMC5504755

[17] Tegnestedt C Günther A Reichard A Bjurström R Alvarsson J Martling C. Levels and sources of sound in the intensive care unit–an observational study of three room types. Acta Anaesth Scand. (2013) 57:1041–50. doi: 10.1111/aas.12138, PMID: 23750596

[18] Luetz A Weiss B Penzel T Fietze I Glos M Wernecke K. Feasibility of noise reduction by a modification in ICU environment. Physiol Meas. (2016) 37:1041–55. doi: 10.1088/0967-3334/37/7/1041, PMID: 27243942

[19] Mac Kenzie D Galbrun L. Noise levels and noise sources in acute care hospital wards. Build Serv Eng Res T. (2007) 28:117–31. doi: 10.1177/0143624406074468

[20] Park M Kohlrausch A Bruijn W, (Eds.), Source-specific analysis of the noise in an intensive care unit. Proceedings of the 42nd international congress and exposition on noise control engineering (2013)

[21] Kahn DM Cook TE Carlisle CC Nelson DL Kramer NR Millman RP. Identification and modification of environmental noise in an ICU setting. Chest. (1998) 114:535–40. doi: 10.1378/chest.114.2.535, PMID: 9726742

[22] Darbyshire JL Muller-Trapet M Cheer J Fazi FM Young JD. Mapping sources of noise in an intensive care unit. Anaesthesia. (2019) 74:1018–25. doi: 10.1111/anae.14690, PMID: 31066046 PMC6767712

[23] Cho OM Kim H Lee YW Cho I. Clinical alarms in intensive care units: perceived obstacles of alarm management and alarm fatigue in nurses. Healthc Inform Res. (2016) 22:46–53. doi: 10.4258/hir.2016.22.1.46, PMID: 26893950 PMC4756058

[24] Petersen EM Costanzo CL. Assessment of clinical alarms influencing Nurses’ perceptions of alarm fatigue. Dimens Crit Care Nurs. (2017) 36:36–44. doi: 10.1097/DCC.0000000000000220, PMID: 27902661

[25] Konkani A Oakley B. Noise in hospital intensive care units—a critical review of a critical topic. J Crit Care. (2012) 27:522.e1. doi: 10.1016/j.jcrc.2011.09.003, PMID: 22033048

[26] Naef AC Knobel SE Ruettgers N Jeitziner M-M Mg H Zante B. Methods for measuring and identifying sounds in the intensive care unit. Front Med. (2022) 9:836203. doi: 10.3389/fmed.2022.836203, PMID: 35733869 PMC9207602

[27] Wallis R Harris E Lee H Davies W Astin F. Environmental noise levels in hospital settings: a rapid review of measurement techniques and implementation in hospital settings. Noise Health. (2019) 21:200–16. doi: 10.4103/nah.NAH_19_18, PMID: 32820743 PMC7650850

[28] Simons KS Park M Kohlrausch A van den Boogaard M Pickkers P de Bruijn W. Noise pollution in the ICU: time to look into the mirror. Crit Care. (2014) 18:1–2. doi: 10.1186/s13054-014-0493-1 PMC4145538 25184539

[29] Delaney L Litton E Van Haren F. The effectiveness of noise interventions in the ICU. Curr Opin Anaesthesiol. (2019) 32:144–9. doi: 10.1097/ACO.0000000000000708 30817386

[30] Murphy E King EA. Chapter 2- principles of environmental noise, Murphy E King EA, (Eds.) Environmental Noise Pollution. Boston: Elsevier; (2014). p. 9–49

[31] Harrison M. Vehicle refinement: controlling noise and vibration in road vehicles Elsevier (2004).

[32] Institute ANS. American National Standard Specification for sound level meters: ANSI S1. 4A-1985 amendment to ANSI S1 4-1983 Acoustical Society of America (1985).

[33] Vreman J van Loon LM van den Biggelaar W van der Hoeven JG Lemson J van den Boogaard M. Contribution of alarm noise to average sound pressure levels in the ICU: an observational cross-sectional study. Intensive Crit Care Nurs. (2020) 61:102901. doi: 10.1016/j.iccn.2020.102901, PMID: 32660883

[34] Darbyshire JL Young JD. An investigation of sound levels on intensive care units with reference to the WHO guidelines. Crit Care. (2013) 17:1–8. doi: 10.1186/cc12870 PMC4056361 24005004

[35] Ryherd EE Waye KP Ljungkvist L. Characterizing noise and perceived work environment in a neurological intensive care unit. J Acoust Soc Am. (2008) 123:747–56. doi: 10.1121/1.2822661, PMID: 18247879

[36] Abatement USOoN. Information on levels of environmental noise requisite to protect public health and welfare with an adequate margin of safety US Government Printing Office (1974).

[37] Erne K Knobel SEJ Naef AC Gerber SM Fischer T Mast FW. Influence of noise manipulation on retention in a simulated ICU ward round: an experimental pilot study. Intensive Care Med Exp. (2022) 10:3. doi: 10.1186/s40635-022-00430-1, PMID: 35089432 PMC8799802

[38] Corey RM Jones U Singer AC. Acoustic effects of medical, cloth, and transparent face masks on speech signals. J Acoust Soc Am. (2020) 148:2371–5. doi: 10.1121/10.0002279, PMID: 33138498 PMC7857499

[39] Passchier-Vermeer W Passchier WF. Noise exposure and public health. Environ Health Perspect. (2000) 108:123–31. doi: 10.1289/ehp.00108s1123, PMID: 10698728 PMC1637786

[40] Ruettgers N Naef AC Rossier M Knobel SE Jeitziner M-M Grosse Holtforth M. Perceived sounds and their reported level of disturbance in intensive care units: a multinational survey among healthcare professionals. PLoS One. (2022) 17:e0279603. doi: 10.1371/journal.pone.0279603, PMID: 36584079 PMC9803129

[41] Chang VA Owens RL LaBuzetta JN. Impact of sleep deprivation in the neurological intensive care unit: a narrative review. Neurocrit Care. (2020) 32:596–608. doi: 10.1007/s12028-019-00795-4, PMID: 31410770 PMC7222162

[42] Delaney LJ Van Haren F Lopez V. Sleeping on a problem: the impact of sleep disturbance on intensive care patients - a clinical review. Ann Intensive Care. (2015) 5:3. doi: 10.1186/s13613-015-0043-2, PMID: 25852963 PMC4385145

[43] Salas RE Gamaldo CE. Adverse effects of sleep deprivation in the ICU. Crit Care Clin. (2008) 24:461–76. doi: 10.1016/j.ccc.2008.02.006 18538195

