# Supplementary material for: Characterization of sound pressure levels and sound sources in the intensive care unit: a 1 week observational study
Source: Front Med (Lausanne). 2023 Jul 13;10:1219257. doi: 10.3389/fmed.2023.1219257 (PMC10382019; doi:10.3389/fmed.2023.1219257)
Supplement: Supplementary file 3 [file Data_Sheet_3.pdf]

Supplementary Material

1 Supplementary Results and Tables

1.1 Supplementary Results

Supplementary Results 1: Sound Pressure Levels

Week 1: L_{AFmax}

The mean of the 8-hour maximum sound pressure level ($L_{AFmax, 8h}$) reached during the day shifts was 69.8 ± 3.7 dBA and 71.4 ± 3.8 dBA for beds 1 and 2, respectively (Table 1). During the evening shifts, the $L_{AFmax, 8h}$ was 73.1 ± 3.8 dBA for bed 1, and 70.9 ± 2.8 dBA for bed 2 (Table 1). The maximum level reached differed significantly based on location during both the day (78.0 ± 3.6 dBA; $F(3, 28) = 9.39$, $p < .001$) and evening (79.4 ± 5.4 dBA; $F(3, 24) = 5.70$, $p < .00429$) shifts (Table 1). Tukey's HSD for multiple comparisons found that the maximum reached in the hallway was significantly greater than at bed 1 (Day: $p < .001$, 95% CI = [-12.786, -3.659]; Evening: $p = .0346$, 95% CI = [-12.199, -0.364]), and bed 2 (Day: $p <.001$, 95% CI = [-11.199, -2.071]; Evening: $p = .00316$, 95% CI = [-14.385, -2.549]) during both the day and evening shifts. The maximum reached in the hallway was also significantly greater at bed A than in the hallway during the day shift, while only a trend was present in the evening shift (Day: $p < .00427$, 95% CI = [-10.836, -1.709]; Evening: $p = .0511$, 95% CI = [-11.813, 0.022]). No significant differences $F(3, 24) = 0.33$, $p = .806$) in the $L_{AFmax, 8h}$ between the different device locations were found during the night shift.

Week 1: L_{AFmin}

The mean of the 8-hour minimum sound pressure level ($L_{AFmin, 8h}$) reached during the day shifts was 41.7 ± 1.4 dBA and 38.6 ± 1.4 dBA for beds 1 and 2, respectively (Table 1). During the evening shifts, the $L_{AFmin, 8h}$ was 41.3 ± 2.3 dBA for bed 1, and 38.8 ± 2.9 dBA for bed 2 (Table 1). During the night shifts, the $L_{AFmin, 8h}$ was 41.0 ± 2.2 dBA for bed 1 and 38.0 ± 2.2 dBA for bed 2 (Table 1). The minimum level reached differed significantly based on location during the day ($F(3, 28) = 26.90$, $p < .001$), evening ($F(3, 24) = 7.39$, $p < .001$), and night ($F(3, 24) = 8.46$, $p < .001$) shift. Tukey's HSD for multiple comparisons found that during the day shift, the minimum reached at bed 2 was significantly lower than at bed 1 ($p < .001$, 95% CI = [1.237, 4.963]) and bed A ($p < .001$, 95% CI = [3.087, 6.813]). The minimum reached in the hallway was also significantly lower than at bed 1 ($p <.001$, 95% CI = [1.446, 5.172]) and bed A ($p < .001$, 95% CI = [3.296, 7.022]) during the day shift. During the evening shift, the minimum level reached was significantly lower at bed 2 ($p < .0069$, 95% CI = [1.027, 7.572]) and in the hallway ($p < .00427$, 95% CI = [1.651, 8.195]), than at bed A.

Supplementary Results 2. Restorative Periods.

When not accounting for bed occupancy, the total number of restorative periods ($L_{Aeq, 5min} < 50$ dB(A)) that occurred during week 1 was 975 (81 h and 15 min) for bed 1 and 1351 (112 h and 35 min) for bed 2. Bed 1 had 141, 309, and 525 restorative periods for the day, evening, and night shift, respectively. Bed 2 had 301, 439, and 611 restorative periods for the same shifts. The mean number of restorative periods during the day shift was 2.27 ± 3.42 (0 h and 11 min) for bed 1 and 4.07 ± 4.89 (0 h and 20 min) for bed 2. The evening shift had a mean of 3.86 ± 9.0 restorative periods (0 h and 19 min) for bed 1 and 5.63 ± 9.75 (0 h and 28 min) for bed 2. The night shift had a mean of 8.47 ± 15.6 (0 h and 42 min) restorative periods for bed 1 and 15.3 ± 19.9 (1 h and 16 min) for bed 2.

1.2 Supplementary Tables

Supplementary Table 1. Categories of Sound Sources for Analysis. Sound sources are grouped for ease of analysis. Categories can be adapted depending how detailed of an analysis is desired. Full details about the categorizations and abbreviations can be found in Naef et al. (26). Only the sound sources that have occurred during the one-week observation period are mentioned. An asterisk (*) indicates the short-lasting sound sources.

		Categories	Included sound sources (abbreviation)
Human (-human interaction) sounds	A1. Verbal		
		Staff < 3 people talking (out of ward round)	*S, E, Shout, SPhone, SS, SPh, SE, EE*
		Staff ≥ 3 people talking (out of ward round)	*SSS, SSPh, SSSS, 5S, 6S, 7S, …*
		Staff during ward round < 3 people talking	*Round S, Round SS*
		Staff during ward round ≥ 3 people talking	*Round SSS, Round SSSS, Round 5S, …*
		Staff talking with patient	*SPat, PhPat, SSPat, …*
		Visitors, staff and patient talking < 3 people	*VV, SV, VPat, PatPhone, …*
		Visitors, staff and patient talking ≥ 3 people	*VVPat, VV, SVV, SSVV, SVVPat, SSVVPat, …*
	A2. Non-verbal		
		Staff sounds	*S-sound, S-cough/sneeze*
		Patient sounds	*Pat-sound, Pat-stuff/bag*
		Visitor sounds	*V-sound*
Object (-human interaction) sounds	B1. Patient care		
		Admission and discharge	*Admission, Discharge*
		Activity of daily living:<br>- Non-mobilization	*Int. washing, Int. water spray, Int. feeding, Int. bedpan/urinal*
		Activity of daily living:<br>- Mobilization	*Int. mobilizing, Int. physiotherapy*
		Nursing	*Int. changing bedsheet, Int. wound care, Int. bandage, Int. death care*
		Diagnostic:<br>- Standard monitoring	*Int. temperature, Int. blood pressure, Int. ECG*
		Diagnostic:<br>- Radiological diagnostic	*Int. X-ray, Int. sonography, Int. doppler*
		Diagnostic:<br>- Neurological interventions	*Int. EEG, Int. EMG, Int. EVD*

	Ventilatory interventions:<br>- Intubation and extubation<br>- Bronchoscopic interventions	 *Int. suctioning, Int. intubation, Int. extubation*<br>*Int. thoracic drain, Int. bronchoscopy, Int. BAL*
	Cardiovascular interventions	*Int. CPR, Int. ECMO, Int. water seal drain (cardiotomy reservoir), Int. central venous catheter, Int. IPC*
	Renal intervention	*Int. RRT*
	Unknown intervention	*Int. unknown*
B2. General activities		
	Preparation board	*Prep1, Prep2, Water*
	Fee standing equipment	*Equip, Supply, Computer*
	Medical Pendant	*Pendant, Hemosphere*
	Bed-related	*Bed*
	Privacy screens	*Door, Divider*
	Short-lasting activities*	*Bump*, Drop*, Chair**
B3. Maintenance		
	Continuous maintenance	*Prepare, Empty, Clean*
	Short-lasting maintenance*	*Lid*, Laundry*, Trash**
B4. Personal items		
	Clothing accessories	*Rattle, Shoes*
	Ringing*	*Pager*, Phone*, Mobile**
B5. Observer		
	Observer talking	*SObserver*
	Observer sounds*	*Chair*, Observer cough/sneeze*
C. Alarms		
	Monitor alarms	

Supplementary Table 2. L_{AFmax} Sound Pressure Levels per Shift and Day. Summary of the L_{AFmax} per shift (8 h) per day in A-weighted decibels for week 1. The total per shift is calculated as the mean and standard deviation (std) across the days. L_{AFmax}, fast A-weighted maximum sound pressure level; hallway, class I sound level meter placed at the nursing station; beds 1, 2, and A, class II sound level meter placed above each bed (26). *Not an entire shift: from study start at 9:15 a.m. to shift end at 3:00 p.m.; **Not an entire shift: from shift start at 7:00 a.m. to study end at 9:15 a.m.

		$L_{AFmax, 8h}$			
**Shift**	**Day**	**Hallway (dBA)**	**Bed 1 (dBA)**	**Bed 2 (dBA)**	**Bed A (dBA)**
**Day**	**Monday***	77.84	69.00	72.10	71.20
	**Tuesday**	76.50	72.10	70.00	71.50
	**Wednesday**	76.47	76.70	71.20	73.20
	**Thursday**	83.73	67.90	75.00	72.80
	**Friday**	82.86	69.00	72.80	71.60
	**Saturday**	77.32	70.60	75.30	74.70
	**Sunday**	76.85	69.30	71.50	71.60
	**Monday****	72.71	63.90	63.30	67.50
	**Mean ± Std**	78.03±3.60	69.81±3.65	71.40±3.75	71.76±2.08
**Evening**	**Monday**	71.77	68.10	67.80	72.00
	**Tuesday**	85.59	72.30	70.70	75.10
	**Wednesday**	80.32	79.00	75.90	80.60
	**Thursday**	79.48	76.00	72.70	70.40
	**Friday**	80.50	73.40	70.90	71.60
	**Saturday**	85.19	73.80	67.90	70.70
	**Sunday**	72.72	69.00	70.40	73.90
	**Mean ± Std**	79.37±5.43	73.09±3.79	70.90±2.80	73.47±3.57
**Night**	**Monday**	72.20	70.20	69.30	66.80
	**Tuesday**	77.47	67.40	64.20	69.00
	**Wednesday**	71.32	74.80	75.10	68.80
	**Thursday**	70.87	72.70	78.40	76.10
	**Friday**	68.56	68.10	68.10	67.00
	**Saturday**	75.42	80.40	76.80	75.10
	**Sunday**	68.54	63.20	59.20	62.60
	**Mean ± Std**	72.06±3.35	70.97±5.61	70.16±7.04	69.34±4.77

Supplementary Table 3. L_{AFmin} Sound Pressure Levels per Shift and Day. Summary of the L_{AFmin} per shift (8 h) per day in A-weighted decibels for week 1. The total per shift is calculated as the mean and standard deviation (std) across the days. L_{AFmin}, fast A-weighted minimum sound pressure level; hallway, class I sound level meter placed at the nursing station; beds 1, 2, and A, class II sound level meter placed above each bed (26). *Not an entire shift: from study start at 9:15 a.m. to shift end at 3:00 p.m.; **Not an entire shift: from shift start at 7:00 a.m. to study end at 9:15 a.m.

		$L_{AFmin, 8h}$			
**Shift**	**Day**	**Hallway (dBA)**	**Bed 1 (dBA)**	**Bed 2 (dBA)**	**Bed A (dBA)**
**Day**	**Monday***	39.06	41.70	39.10	45.30
	**Tuesday**	38.53	43.40	40.80	44.90
	**Wednesday**	39.38	44.00	40.50	46.20
	**Thursday**	37.76	41.40	37.70	41.20
	**Friday**	38.72	41.60	38.40	42.40
	**Saturday**	37.28	41.50	37.70	43.20
	**Sunday**	37.77	40.00	37.30	42.60
	**Monday****	38.63	40.00	37.30	42.60
	**Mean ± Std**	38.39±0.72	41.70±1.42	38.60±1.40	43.55±1.7
**Evening**	**Monday**	38.49	43.80	43.80	45.50
	**Tuesday**	38.86	42.90	40.80	45.70
	**Wednesday**	38.90	42.10	38.50	40.80
	**Thursday**	39.12	43.70	39.50	45.30
	**Friday**	36.70	39.10	35.60	41.80
	**Saturday**	38.25	38.60	36.00	41.00
	**Sunday**	37.02	39.00	37.50	41.70
	**Mean ± Std**	38.19±0.96	41.31±2.33	38.81±2.87	43.11±2.26
**Night**	**Monday**	37.63	42.90	40.80	45.30
	**Tuesday**	37.37	42.20	40.30	45.40
	**Wednesday**	38.15	41.00	37.00	40.10
	**Thursday**	39.19	42.70	38.50	45.50
	**Friday**	37.83	42.40	38.80	42.60
	**Saturday**	36.94	37.90	35.20	39.50
	**Sunday**	38.05	38.10	35.50	39.80
	**Mean ± Std**	37.88±0.71	41.03±2.16	38.01±2.20	42.60±2.81

Supplementary Table 4. Bed occupancy during study. Bed occupancy presented as a percentage of the maximum capacity per day of the study. "All Beds" represent a maximum of 16 beds spread across the entire ward. Beds 1 and 2 represent the two beds that were the focus of our study.
* Not an entire shift: from study start at 9:15 a.m. to shift end at 3:00 p.m.
** Not an entire shift: from shift start at 7:00 a.m., to study end at 9:15 a.m.

	All Beds		Beds 1 & 2	
**Day**	**Occupied (%)**	**Unoccupied (%)**	**Occupied (%)**	**Unoccupied (%)**
**Monday***	74.79	25.21	85.00	15.00
**Tuesday**	83.33	16.67	100.00	0.00
**Wednesday**	77.47	22.53	88.54	11.46
**Thursday**	71.35	28.65	100.00	0.00
**Friday**	70.70	29.30	73.96	26.04
**Saturday**	78.13	21.88	59.38	40.63
**Sunday**	66.41	33.59	46.88	53.13
**Monday****	73.26	26.74	100.00	0.00

Supplementary Table 5. $L_{Aeq, 1week}$, $L_{AFmax, 1week}$, and $L_{AFmin, 1week}$ between four weeks. Summary of the L_{Aeq}, L_{AFmax}, and L_{AFmin} per week (168 h) per bed for 4 weeks. L_{Aeq}, A-weighted time-averaged sound pressure level; L_{AFmax}, fast A-weighted maximum sound pressure level; L_{AFmin}, fast A-weighted minimum sound pressure level; dBA, A-weighted decibels.

		Week 1	Week 2	Week 3	Week 4
L_{Aeq} (dBA)	Bed 1	51.80	52.45	52.52	51.33
	Bed 2	50.09	51.52	50.82	50.25
L_{AFmax} (dBA)	Bed 1	80.40	80.40	79.60	85.20
	Bed 2	78.40	74.40	82.50	83.30
L_{AFmin} (dBA)	Bed 1	37.90	37.20	38.00	36.70
	Bed 2	35.20	34.70	35.80	34.90

Supplementary Table 6. **Five longest restorative periods per bed.** *WKND*, weekend; *WKDY*, weekday; *Wed*, Wednesday; *Fri*, Friday; *Sat,* Saturday; *Sun*, Sunday; *h*, hours.

Bed	Duration	Weekday	Shift	Bed Occupancy	Start Time	End Time
1	12 h 30 min	WKND	Evening & night	Unoccupied	Sat 04:30 p.m.	05:00 a.m.
1	7 h 50 min	WKND	Night	Occupied	Sun11:00 p.m.	06:50 a.m.
1	2 h 35 min	WKND	Night	Occupied	Sat 04:10 a.m.	06:45 a.m.
1	2 h 30 min	WKDY	Night	Occupied	Wed 11:25 p.m.	01:55 a.m.
1	2 h 10 min	WKND	Day	Unoccupied	Sun 12:00 p.m.	14:10 p.m.
2	12 h 30 min	WKND	Evening & night	Unoccupied	Sat 04:30 p.m.	05:00 a.m.
2	10 h 40 min	WKND	Evening & night	Occupied	Sun 08:30 p.m.	07:10 a.m.
2	4 h 30 min	WKDY	Night	Occupied	Wed 00:25 a.m.	04:55 a.m.
2	4 h 5 min	WKDY	Night	Occupied	Fri 01:00 a.m.	05:05 a.m.
2	2 h 35 min	WKND	Night	Occupied	Sat 04:10 a.m.	06:45 a.m.

Supplementary Table 7. Sound source results for all categories, all days. All days: Monday, August 23rd until Monday, August 30th (09:15 a.m. - 09:15 a.m.). Total time was 168 h minus observer breaks, total = 140 h. Where there is no minute or percent value given, the number represents the number of occurrences. N/A indicates no occurrence of the category at that bed. Overall = 8400 min; Day, Evening, Night = 2800 min.

	Description	Bed 1				Bed 2			
		**Overall**	**Day**	**Evening**	**Night**	**Overall**	**Day**	**Evening**	**Night**
**Human (-Human) Sounds**	**Staff < 3 people talking (out of ward round)**	2248 min<br>26.76 %	720 min<br>25.71 %	740 min<br>26.43 %	788 min<br>28.14 %	1536 min<br>18.29 %	769 min<br>27.46 %	503 min<br>17.96 %	264 min<br>9.43 %
	**Staff ≥ 3 people talking (out of ward round)**	370 min<br>4.40 %	134 min<br>4.79 %	139 min<br>4.96 %	97 min<br>3.46 %	346 min<br>4.12 %	186 min<br>6.64 %	103 min<br>3.68 %	57 min<br>2.04 %
	**Staff during ward round < 3 people talking**	39 min<br>0.46 %	11 min<br>0.39 %	28 min<br>1.00 %	0 min<br>0.00%	36 min<br>0.43 %	20 min<br>0.71 %	15 min<br>0.54 %	1 min<br>0.04 %
	**Staff during ward round ≥ 3 people talking**	49 min<br>0.58 %	24 min<br>0.86 %	17 min<br>0.61 %	8 min<br>0.29 %	30 min<br>0.36 %	24 min<br>0.86 %	6 min<br>0.21 %	0 min<br>0.00 %
	**Staff talking with patient**	833 min<br>9.92 %	247 min<br>8.82 %	308 min<br>11.00 %	278 min<br>9.93 %	948 min<br>11.29 %	431 min<br>15.39 %	248 min<br>8.86 %	269 min<br>9.61 %
	**Visitors, staff and patient talking < 3 people**	61 min<br>0.73 %	47 min<br>1.68 %	14 min<br>0.50 %	0 min<br>0.00 %	97 min<br>1.15 %	37 min<br>1.32 %	57 min<br>2.04 %	3 min<br>0.11 %
	**Visitors, staff and patient talking ≥ 3 people**	359 min<br>4.27 %	133 min<br>4.75 %	226 min<br>8.07 %	0 min<br>0.00 %	14 min<br>0.17 %	0 min<br>0.00 %	1 min<br>0.04 %	13 min<br>0.46 %
	**Staff sounds**	20 min<br>0.24 %	5 min<br>0.18 %	15 min<br>0.54 %	0 min<br>0.00 %	40 min<br>0.48 %	14 min<br>0.50 %	22 min<br>0.79 %	4 min<br>0.14 %
	**Patient sounds**	176 min<br>2.10 %	91 min<br>3.25 %	18 min<br>0.64 %	67 min<br>2.39 %	445 min<br>5.30 %	195 min<br>6.96 %	91 min<br>3.25 %	159 min<br>5.68 %
	**Visitor sounds**	6 min<br>0.07 %	6 min<br>0.21 %	0 min<br>0.00 %	0 min<br>0.00 %	N/A	N/A	N/A	N/A
**Object (-Human Interaction) Sounds**	**Admission and discharge**	13 min<br>0.15 %	4 min<br>0.14 %	9 min<br>0.32 %	0 min<br>0.00 %	109 min<br>1.30 %	53 min<br>1.89 %	43 min<br>1.54 %	13 min<br>0.46 %
	**Activity of daily living: Non-mobilization**	66 min<br>0.79 %	34 min<br>1.21 %	22 min<br>0.79 %	10 min<br>0.36 %	103 min<br>1.23 %	73 min<br>2.61 %	17 min<br>0.61 %	13 min<br>0.46 %
	**Activity of daily living: Mobilization**	89 min<br>1.06 %	46 min<br>1.64 %	21 min<br>0.75 %	22 min<br>0.79 %	27 min<br>0.32 %	14 min<br>0.50 %	7 min<br>0.25 %	6 min<br>0.21 %
	**Nursing**	224 min<br>2.67 %	46 min<br>1.64 %	176 min<br>6.29 %	2 min<br>0.07 %	43 min<br>0.51 %	14 min<br>0.50 %	6 min<br>0.21 %	23 min<br>0.82 %

	**Diagnostic: Standard monitoring**	22 min 0.26 %	0 min 0.00 %	11 min 0.39 %	11 min 0.39 %	28 min 0.33 %	13 min 0.46 %	9 min 0.32 %	6 min 0.21 %
	**Diagnostic: Radiological diagnostic**	34 min 0.40 %	14 min 0.50 %	20 min 0.71 %	0 min 0.00 %	143 min 1.70 %	122 min 4.36 %	20 min 0.71 %	1 min 0.04 %
	**Diagnostic: Neurological interventions**	N/A	N/A	N/A	N/A	29 min 0.35 %	29 min 1.04 %	0 min 0.00 %	0 min 0.00 %
	**Ventilatory interventions: Intubation and extubation + Bronchoscopic interventions**	1263 min 15.04 %	226 min 8.07 %	543 min 19.39 %	494 min 17.64 %	365 min 4.35 %	16 min 0.57 %	189 min 6.75 %	160 min 5.71 %
	**Cardiovascular interventions**	6 min 0.07 %	0 min 0.00 %	0 min 0.00 %	6 min 0.21 %	2064 min 24.57 %	338 min 12.07 %	1072min 38.29 %	654 min 23.36 %
	**Renal intervention**	N/A	N/A	N/A	N/A	279 min 3.32 %	192 min 6.86 %	87 min 3.11 %	0 min 0.00 %
	**Unknown intervention**	26 min 0.31 %	2 min 0.07 %	24 min 0.86 %	0 min 0.00 %	7 min 0.08 %	6 min 0.21 %	1 min 0.04 %	0 min 0.00 %
	**Preparation board**	1780 min 21.19 %	557 min 19.89 %	611 min 21.82 %	612 min 21.86 %	539 min 6.42 %	301 min 10.75 %	155 min 5.54 %	83 min 2.96 %
	**Free standing equipment**	549 min 6.54 %	144 min 5.14 %	199 min 7.11 %	206 min 7.36 %	604 min 7.19 %	273 min 9.75 %	144 min 5.14 %	187 min 6.68 %
	**Pendant**	1157 min 13.77 %	407 min 14.54 %	441 min 15.75 %	309 min 11.04 %	1066 min 12.63 %	440 min 15.71 %	365 min 13.04 %	261 min 9.32 %
	**Bed-related**	166 min 1.98 %	72 min 2.57 %	69 min 2.46 %	25 min 0.89 %	238 min 2.83 %	130 min 4.64 %	73 min 2.61 %	35 min 1.25 %
	**Privacy screens**	74 min 0.88 %	26 min 0.93 %	39 min 1.39 %	9 min 0.32 %	59 min 0.70 %	40 min 1.43 %	18 min 0.64 %	1 min 0.04 %
	**Short-lasting activities***	538 x	257 x	224 x	57 x	478 x	236 x	207 x	35 x
	**Continuous maintenance**	104 min 1.24 %	64 min 2.29 %	18 min 0.64 %	22 min 0.79 %	160 min 1.90 %	109 min 3.89 %	47 min 1.68 %	4 min 0.14 %
	**Short-lasting maintenance***	528 x	233 x	219 x	76 x	399 x	213 x	135 x	51 x
	**Clothing accessories**	845 min 10.06 %	287 min 10.25 %	362 min 12.93 %	196 min 7.00 %	561 min 6.68 %	271 min 9.68 %	185 min 6.61 %	105 min 3.75 %
	**Ringing***	65 x	19 x	30 x	16 x	186 x	106 x	65 x	15 x
**Observer**	**Observer talking**	N/A	N/A	N/A	N/A	92 min 1.10 %	53 min 1.89 %	32 min 1.14 %	7 min 0.25 %
	**Observer-sound***	N/A	N/A	N/A	N/A	23 x	10 x	11 x	2 x

Supplementary Table 8. Sound source results for all categories, day 1. Monday, August 23rd (09:15 a.m. - 07:00 a.m.). Total time was 21 h 45 min minus observer breaks, total = 18 h. Where there is no minute or percent value given, the number represents the number of occurrences. N/A indicates no occurrence of the category at that bed. Overall = 1085 min; *Day = 285 min (2.25 hours less than other days); Evening, Night = 400 min.

	Description	Bed 1				Bed 2			
		Overall	Day*	Evening	Night	Overall	Day*	Evening	Night
**Human (-Human) Sounds**	**Staff < 3 people talking (out of ward round)**	288 min 26.54 %	66 min 23.16 %	123 min 30.75 %	99 min 24.75 %	264 min 24.33 %	73 min 25.61 %	126 min 31.50 %	65 min 16.25 %
	**Staff ≥ 3 people talking (out of ward round)**	52 min 4.79 %	18 min 6.32 %	10 min 2.50 %	24 min 6.00 %	44 min 4.06 %	6 min 2.11 %	30 min 7.50 %	8 min 2.00 %
	**Staff during ward round < 3 people talking**	7 min 0.65 %	0 min 0.00 %	7 min 1.75 %	0 min 0.00 %	5 min 0.46 %	0 min 0.00 %	5 min 1.25 %	0 min 0.00 %
	**Staff during ward round ≥ 3 people talking**	N/A	N/A	N/A	N/A	3 min 0.28 %	3 min 1.05 %	0 min 0.00 %	0 min 0.00 %
	**Staff talking with patient**	128 min 11.80 %	5 min 1.75 %	68 min 17.00 %	55 min 13.75 %	117 min 10.78 %	29 min 10.18 %	13 min 3.25 %	75 min 18.75 %
	**Visitors, staff and patient talking < 3 people**	N/A	N/A	N/A	N/A	3 min 0.28 %	3 min 1.05 %	0 min 0.00 %	0 min 0.00 %
	**Visitors, staff and patient talking ≥ 3 people**	31 min 2.86 %	0 min 0.00 %	31 min 7.75 %	0 min 0.00 %	N/A	N/A	N/A	N/A
	**Staff sounds**	2 min 0.18 %	0 min 0.00 %	2 min 0.50 %	0 min 0.00 %	2 min 0.18 %	0 min 0.00 %	1 min 0.25 %	1 min 0.25 %
	**Patient sounds**	N/A	N/A	N/A	N/A	43 min 3.96 %	38 min 13.33 %	0 min 0.00 %	5 min 1.25 %
	**Visitor sounds**	N/A	N/A	N/A	N/A	N/A	N/A	N/A	N/A
**Object (-Human Interaction) Sounds**	**Admission and discharge**	4 min 0.37 %	4 min 1.40 %	0 min 0.00 %	0 min 0.00 %	27 min 2.49 %	5 min 1.75 %	22 min 5.50 %	0 min 0.00 %
	**Activity of daily living: Non-mobilization**	7 min 0.65 %	0 min 0.00 %	7 min 1.75 %	0 min 0.00 %	2 min 0.18 %	0 min 0.00 %	0 min 0.00 %	2 min 0.50 %
	**Activity of daily living: Mobilization**	9 min 0.83 %	0 min 0.00 %	5 min 1.25 %	4 min 1.00 %	2 min 0.18 %	0 min 0.00 %	0 min 0.00 %	2 min 0.50 %
	**Nursing**	3 min 0.28 %	1 min 0.35 %	2 min 0.50 %	0 min 0.00 %	13 min 1.20 %	0 min 0.00 %	0 min 0.00 %	13 min 3.25 %

	**Diagnostic: Standard monitoring**	N/A	N/A	N/A	N/A	1 min<br>0.09 %	0 min<br>0.00 %	0 min<br>0.00 %	1 min<br>0.25 %
	**Diagnostic: Radiological diagnostic**	N/A	N/A	N/A	N/A	10 min<br>0.92 %	0 min<br>0.00 %	9 min<br>2.25 %	1 min<br>0.25 %
	**Diagnostic: Neurological interventions**	N/A	N/A	N/A	N/A	N/A	N/A	N/A	N/A
	**Ventilatory interventions: Intubation and extubation + Bronchoscopic interventions**	30 min<br>2.76 %	2 min<br>0.70 %	16 min<br>4.00 %	12 min<br>3.00 %	10 min<br>0.92 %	0 min<br>0.00 %	0 min<br>0.00 %	10 min<br>2.50 %
	**Cardiovascular interventions**	N/A	N/A	N/A	N/A	660 min<br>60.83 %	0 min<br>0.00 %	360 min<br>90.00 %	300 min<br>75.00 %
	**Renal intervention**	N/A	N/A	N/A	N/A	N/A	N/A	N/A	N/A
	**Unknown intervention**	N/A	N/A	N/A	N/A	N/A	N/A	N/A	N/A
	**Preparation board**	245 min<br>22.58 %	54 min<br>18.95 %	98 min<br>24.50 %	93 min<br>23.25 %	83 min<br>7.65 %	31 min<br>10.88 %	25 min<br>6.25 %	27 min<br>6.75 %
	**Free standing equipment**	47 min<br>4.33 %	12 min<br>4.21 %	13 min<br>3.25 %	22 min<br>5.50 %	73 min<br>6.73 %	27 min<br>9.47 %	11 min<br>2.75 %	35 min<br>8.75 %
	**Pendant**	139 min<br>12.81 %	47 min<br>16.49 %	51 min<br>12.75 %	41 min<br>10.25 %	190 min<br>17.51 %	34 min<br>11.93 %	77 min<br>19.25 %	79 min<br>19.75 %
	**Bed-related**	29 min<br>2.67 %	6 min<br>2.11 %	18 min<br>4.50 %	5 min<br>1.25 %	39 min<br>3.59 %	12 min<br>4.21 %	16 min<br>4.00 %	11 min<br>2.75 %
	**Privacy screens**	5 min<br>0.46 %	1 min<br>0.35 %	3 min<br>0.75 %	1 min<br>0.25 %	2 min<br>0.18 %	1 min<br>0.35 %	1 min<br>0.25 %	0 min<br>0.00 %
	**Short-lasting activities***	30 x	11 x	8 x	11 x	27 x	9 x	10 x	8 x
	**Continuous maintenance**	19 min<br>1.75 %	14 min<br>4.91 %	0 min<br>0.00 %	5 min<br>1.25 %	27 min<br>2.49 %	25 min<br>8.77 %	0 min<br>0.00 %	2 min<br>0.50 %
	**Short-lasting maintenance***	72 x	22 x	35 x	15 x	39 x	10 x	20 x	9 x
	**Clothing accessories**	86 min<br>7.93 %	36 min<br>12.63 %	21 min<br>5.25 %	29 min<br>7.25 %	82 min<br>7.56 %	23 min<br>8.07 %	15 min<br>3.75 %	44 min<br>11.00 %
	**Ringing***	5 x	0 x	1 x	4 x	20 x	12 x	6 x	2 x
**Observer**	**Observer talking**	N/A	N/A	N/A	N/A	13 min<br>1.20 %	10 min<br>3.51 %	3 min<br>0.75 %	0 min<br>0.00 %
	**Observer sound***	N/A	N/A	N/A	N/A	1 x	0 x	1 x	0 x

Supplementary Table 9. Sound source results for all categories, day 2. Tuesday, August 24th (07:00 a.m. - 07:00 a.m.). Total time was 24 h minus observer breaks, total = 20 h. Where there is no minute or percent value given, the number represents the number of occurrences. N/A indicates no occurrence of the category at that bed. Overall = 1200 min; Day, Evening, Night = 400 min.

	Description	Bed 1				Bed 2			
		**Overall**	**Day**	**Evening**	**Night**	**Overall**	**Day**	**Evening**	**Night**
**Human (-Human) Sounds**	**Staff < 3 people talking (out of ward round)**	275 min 22.92 %	80 min 20.00 %	138 min 34.50 %	57 min 14.25 %	278 min 23.17 %	169 min 42.25 %	101 min 25.25 %	8 min 2.00 %
	**Staff ≥ 3 people talking (out of ward round)**	64 min 5.33 %	28 min 7.00 %	36 min 9.00 %	0 min 0.00 %	89 min 7.42 %	78 min 19.50 %	11 min 2.75 %	0 min 0.00 %
	**Staff during ward round < 3 people talking**	3 min 0.25 %	0 min 0.00 %	3 min 0.75 %	0 min 0.00 %	4 min 0.33 %	1 min 0.25 %	3 min 0.75 %	0 min 0.00 %
	**Staff during ward round ≥ 3 people talking**	7 min 0.58 %	5 min 1.25 %	2 min 0.50 %	0 min 0.00 %	7 min 0.58 %	5 min 1.25 %	2 min 0.50 %	0 min 0.00 %
	**Staff talking with patient**	127 min 10.58 %	40 min 10.00 %	44 min 11.00 %	43 % 10.75 %	111 min 9.25 %	51 min 12.75 %	44 min 11.00 %	16 min 4.00 %
	**Visitors, staff and patient talking < 3 people**	12 min 1.00 %	0 min 0.00 %	12 min 3.00 %	0 min 0.00 %	N/A	N/A	N/A	N/A
	**Visitors, staff and patient talking ≥ 3 people**	48 min 4.00 %	0 min 0.00 %	48 min 12.00 %	0 min 0.00 %	N/A	N/A	N/A	N/A
	**Staff sounds**	4 min 0.33 %	0 min 0.00 %	4 min 1.00 %	0 min 0.00 %	14 min 1.17 %	1 min 0.25 %	12 min 3.00 %	1 min 0.25 %
	**Patient sounds**	23 min 1.92 %	0 min 0.00 %	4 min 1.00 %	19 min 4.75 %	53 min 4.42 %	25 min 6.25 %	9 min 2.25 %	19 min 4.75 %
	**Visitor sounds**	N/A	N/A	N/A	N/A	N/A	N/A	N/A	N/A
**Object (-Human Interaction) Sounds**	**Admission and discharge**	N/A	N/A	N/A	N/A	19 min 1.58 %	19 min 4.75 %	0 min 0.00 %	0 min 0.00 %
	**Activity of daily living: Non-mobilization**	6 min 0.50 %	3 min 0.75 %	1 min 0.25 %	2 min 0.50 %	11 min 0.92 %	8 min 2.00 %	3 min 0.75 %	0 min 0.00 %
	**Activity of daily living: Mobilization**	6 min 0.50 %	4 min 1.00 %	0 min 0.00 %	2 min 0.50 %	2 min 0.17 %	0 min 0.00 %	2 min 0.50 %	0 min 0.00 %
	**Nursing**	4 min 0.33 %	0 min 0.00 %	2 min 0.50 %	2 min 0.50 %	8 min 0.67 %	7 min 1.75 %	1 min 0.25 %	0 min 0.00 %
	**Diagnostic: Standard monitoring**	11 min 0.92 %	0 min 0.00 %	1 min 0.25 %	10 min 2.50 %	3 min 0.25 %	0 min 0.00 %	3 min 0.75 %	0 min 0.00 %

	**Diagnostic: Radiological diagnostic**	29 min 2.42 %	9 min 2.25 %	20 min 5.00 %	0 min 0.00 %	120 min 10.00 %	109 min 27.25 %	11 min 2.75 %	0 min 0.00 %
	**Diagnostic: Neurological interventions**	N/A	N/A	N/A	N/A	N/A	N/A	N/A	N/A
	**Ventilatory interventions: Intubation and extubation + Bronchoscopic interventions**	283 min 23.58 %	19 min 4.75 %	144 min 36.00 %	120 min 30.00 %	262 min 21.83 %	3 min 0.75 %	139 min 34.75 %	120 min 30.00 %
	**Cardiovascular interventions**	N/A	N/A	N/A	N/A	70 min 5.83 %	70 min 17.50 %	0 min 0.00 %	0 min 0.00 %
	**Renal intervention**	N/A	N/A	N/A	N/A	34 min 2.83 %	33 min 8.25 %	1 min 0.25 %	0 min 0.00 %
	**Unknown intervention**	18 min 1.50 %	0 min 0.00 %	18 min 4.50 %	0 min 0.00 %	2 min 0.17 %	1 min 0.25 %	1 min 0.25 %	0 min 0.00 %
	**Preparation board**	275 min 22.92 %	84 min 21.00 %	113 min 28.25 %	78 min 19.50 %	105 min 8.75 %	63 min 15.75 %	37 min 9.25 %	5 min 1.25 %
	**Free standing equipment**	89 min 7.42 %	36 min 9.00 %	37 min 9.25 %	16 min 4.00 %	130 min 10.83 %	68 min 17.00 %	57 min 14.25 %	5 min 1.25 %
	**Pendant**	182 min 15.17 %	59 min 14.75 %	80 min 20.00 %	43 min 10.75 %	210 min 17.50 %	92 min 23.00 %	102 min 25.50 %	16 min 4.00 %
	**Bed-related**	38 min 3.17 %	9 min 2.25 %	23 min 5.75 %	6 min 1.50 %	49 min 4.08 %	33 min 8.25 %	16 min 4.00 %	0 min 0.00 %
	**Privacy screens**	11 min 0.92 %	2 min 0.50 %	7 min 1.75 %	2 min 0.50 %	1 min 0.08 %	0 min 0.00 %	1 min 0.25 %	0 min 0.00 %
	**Short-lasting activities***	98 x	28 x	48 x	22 x	102 x	47 x	46 x	9 x
	**Continuous maintenance**	14 min 1.17 %	9 min 2.25 %	0 min 0.00 %	5 min 1.25 %	16 min 1.33 %	15 min 3.75 %	1 min 0.25 %	0 min 0.00 %
	**Short-lasting maintenance***	97 x	48 x	42 x	7 x	105 x	64 x	40 x	1 x
	**Clothing accessories**	127 min 10.58 %	57 min 14.25 %	39 min 9.75 %	31 min 7.75 %	147 min 12.25 %	83 min 20.75 %	51 min 12.75 %	13 min 3.25 %
	**Ringing***	13 x	5 x	6 x	2 x	26 x	17 x	9 x	0 x
**Observer**	**Observer talking**	N/A	N/A	N/A	N/A	17 min 1.42 %	12 min 3.00 %	4 min 1.00 %	1 min 0.25 %
	**Observer sound***	N/A	N/A	N/A	N/A	3 x	1 x	2 x	0 x

Supplementary Table 10. Sound source results for all categories, day 3. Wednesday, August 25th (07:00 a.m. - 07:00 a.m.). Total time was 24 h minus observer breaks, total = 20 h. Where there is no minute or percent value given, the number represents the number of occurrences. N/A indicates no occurrence of the category at that bed. Overall = 1200 min; Day, Evening, Night = 400 min.

	Description	Bed 1				Bed 2			
		Overall	Day	Evening	Night	Overall	Day	Evening	Night
Human (-Human) Sounds	Staff < 3 people talking (out of ward round)	395 min 32.92 %	146 min 36.50 %	106 min 26.50 %	143 min 35.75 %	243 min 20.25 %	100 min 25.00 %	75 min 18.75 %	68 min 17.00 %
	Staff ≥ 3 people talking (out of ward round)	56 min 4.67 %	18 min 4.50 %	9 min 2.25 %	29 min 7.25 %	49 min 4.08 %	5 min 1.25 %	22 min 5.50 %	22 min 5.50 %
	Staff during ward round < 3 people talking	7 min 0.58 %	5 min 1.25 %	2 min 0.50 %	0 min 0.00 %	8 min 0.67 %	7 min 1.75 %	1 min 0.25 %	0 min 0.00 %
	Staff during ward round ≥ 3 people talking	6 min 0.50 %	5 min 1.25 %	1 min 0.25 %	0 min 0.00 %	11 min 0.92 %	8 min 2.00 %	3 min 0.75 %	0 min 0.00 %
	Staff talking with patient	291 min 24.25 %	129 min 32.25 %	89 min 22.25 %	73 min 18.25 %	209 min 17.42 %	92 min 23.00 %	59 min 14.75 %	58 min 14.50 %
	Visitors, staff and patient talking < 3 people	2 min 0.17 %	0 min 0.00 %	2 min 0.50 %	0 min 0.00 %	25 min 2.08 %	9 min 2.25 %	13 min 3.25 %	3 min 0.75 %
	Visitors, staff and patient talking ≥ 3 people	67 min 5.58 %	0 min 0.00 %	67 min 16.75 %	0 min 0.00 %	13 min 1.08 %	0 min 0.00 %	0 min 0.00 %	13 min 3.25 %
	Staff sounds	5 min 0.42 %	3 min 0.75 %	2 min 0.50 %	0 min 0.00 %	5 min 0.42 %	2 min 0.50 %	3 min 0.75 %	0 min 0.00 %
	Patient sounds	69 min 5.75 %	58 min 14.50 %	9 min 2.25 %	2 min 0.50 %	64 min 5.33 %	40 min 10.00 %	15 min 3.75 %	9 min 2.25 %
	Visitor sounds	N/A	N/A	N/A	N/A	N/A	N/A	N/A	N/A
Object (-Human Interaction) Sounds	Admission and discharge	N/A	N/A	N/A	N/A	23 min 1.92 %	8 min 2.00 %	15 min 3.75 %	0 min 0.00 %
	Activity of daily living: Non-mobilization	10 min 0.83 %	4 min 1.00 %	3 min 0.75 %	3 min 0.75 %	21 min 1.75 %	11 min 2.75 %	0 min 0.00 %	10 min 2.50 %
	Activity of daily living: Mobilization	20 min 1.67 %	11 min 2.75 %	5 min 1.25 %	4 min 1.00 %	N/A	N/A	N/A	N/A
	Nursing	102 min 8.50 %	0 min 0.00 %	102 min 25.50 %	0 min 0.00 %	10 min 0.83 %	0 min 0.00 %	0 min 0.00 %	10 min 2.50 %
	Diagnostic: Standard monitoring	1 min 0.08 %	0 min 0.00 %	0 min 0.00 %	1 min 0.25 %	5 min 0.42 %	0 min 0.00 %	1 min 0.25 %	4 min 1.00 %

	**Diagnostic: Radiological diagnostic**	N/A	N/A	N/A	N/A	N/A	N/A	N/A	N/A
	**Diagnostic: Neurological interventions**	N/A	N/A	N/A	N/A	N/A	N/A	N/A	N/A
	**Ventilatory interventions: Intubation and extubation + Bronchoscopic interventions**	490 min 40.83 %	176 min 44.00 %	189 min 47.25 %	125 min 31.25 %	4 min 0.33 %	1 min 0.25 %	3 min 0.75 %	0 min 0.00 %
	**Cardiovascular interventions**	N/A	N/A	N/A	N/A	176 min 14.67 %	0 min 0.00 %	65 min 16.25 %	111 min 27.75 %
	**Renal intervention**	N/A	N/A	N/A	N/A	245 min 20.42 %	159 min 39.75 %	86 min 21.50 %	0 min 0.00 %
	**Unknown intervention**	N/A	N/A	N/A	N/A	5 min 0.42 %	5 min 1.25 %	0 min 0.00 %	0 min 0.00 %
	**Preparation board**	239 min 19.92 %	77 min 19.25 %	81 min 20.25 %	81 min 20.25 %	92 min 7.67 %	43 min 10.75 %	29 min 7.25 %	20 min 5.00 %
	**Free standing equipment**	110 min 9.17 %	25 min 6.25 %	40 min 10.00 %	45 min 11.25 %	110 min 9.17 %	54 min 13.50 %	36 min 9.00 %	20 min 5.00 %
	**Pendant**	217 min 18.08 %	75 min 18.75 %	80 min 20.00 %	62 min 15.50 %	138 min 11.50 %	54 min 13.50 %	54 min 13.50 %	30 min 7.50 %
	**Bed-related**	50 min 4.17 %	35 min 8.75 %	8 min 2.00 %	7 min 1.75 %	39 min 3.25 %	19 min 4.75 %	11 min 2.75 %	9 min 2.25 %
	**Privacy screens**	9 min 0.75 %	1 min 0.25 %	6 min 1.50 %	2 min 0.50 %	7 min 0.58 %	5 min 1.25 %	2 min 0.50 %	0 min 0.00 %
	**Short-lasting activities***	138 x	56 x	75 x	7 x	54 x	19 x	26 x	9 x
	**Continuous maintenance**	17 min 1.42 %	12 min 3.00 %	0 min 0.00 %	5 min 1.25 %	46 min 3.83 %	13 min 3.25 %	31 min 7.75 %	2 min 0.50 %
	**Short-lasting maintenance***	86 x	29 x	42 x	15 x	73 x	38 x	26 x	9 x
	**Clothing accessories**	126 min 10.50 %	48 min 12.00 %	48 min 12.00 %	30 min 7.50 %	70 min 5.83 %	43 min 10.75 %	18 min 4.50 %	9 min 2.25 %
	**Ringing***	11 x	4 x	5 x	2 x	40 x	22 x	14 x	4 x
**Observer**	**Observer talking**	N/A	N/A	N/A	N/A	17 min 1.42 %	10 min 2.50 %	3 min 0.75 %	4 min 1.00 %
	**Observer sound***	N/A	N/A	N/A	N/A	4 x	2 x	1 x	1 x

Supplementary Table 11. Sound source results for all categories, day 4. Thursday, August 26th (07:00 a.m. - 07:00 a.m.). Total time was 24 h minus observer breaks, total = 20 h. Where there is no minute or percent value given, the number represents the number of occurrences. N/A indicates no occurrence of the category at that bed. Overall = 1200 min; Day, Evening, Night = 400 min.

	Description	Bed 1				Bed 2			
		**Overall**	**Day**	**Evening**	**Night**	**Overall**	**Day**	**Evening**	**Night**
**Human (-Human) Sounds**	**Staff < 3 people talking (out of ward round)**	382 min 31.83 %	105 min 26.25 %	133 min 33.25 %	144 min 36.00 %	236 min 19.67 %	122 min 30.50 %	80 min 20.00 %	34 min 8.50 %
	**Staff ≥ 3 people talking (out of ward round)**	41 min 3.42 %	7 min 1.75 %	24 min 6.00 %	10 min 2.50 %	20 min 1.67 %	14 min 3.50 %	6 min 1.50 %	0 min 0.00 %
	**Staff during ward round < 3 people talking**	11 min 0.92 %	2 min 0.50 %	9 min 2.25 %	0 min 0.00 %	9 min 0.75 %	5 min 1.25 %	4 min 1.00 %	0 min 0.00 %
	**Staff during ward round ≥ 3 people talking**	13 min 1.08 %	6 min 1.50 %	7 min 1.75 %	0 min 0.00 %	1 min 0.08 %	0 min 0.00 %	1 min 0.25 %	0 min 0.00 %
	**Staff talking with patient**	90 min 7.50 %	25 min 6.25 %	37 min 9.25 %	28 min 7.00 %	178 min 14.83 %	77 min 19.25 %	58 min 14.50 %	43 min 10.75 %
	**Visitors, staff and patient talking < 3 people**	N/A	N/A	N/A	N/A	42 min 3.50 %	1 min 0.25 %	41 min 10.25 %	0 min 0.00 %
	**Visitors, staff and patient talking ≥ 3 people**	50 min 4.17 %	0 min 0.00 %	50 min 12.50 %	0 min 0.00 %	1 min 0.08 %	0 min 0.00 %	1 min 0.25 %	0 min 0.00 %
	**Staff sounds**	2 min 0.17 %	2 min 0.50 %	0 min 0.00 %	0 min 0.00 %	4 min 0.33 %	3 min 0.75 %	1 min 0.25 %	0 min 0.00 %
	**Patient sounds**	N/A	N/A	N/A	N/A	26 min 2.17 %	14 min 3.50 %	10 min 2.50 %	2 min 0.50 %
	**Visitor sounds**	N/A	N/A	N/A	N/A	N/A	N/A	N/A	N/A
**Object (-Human Interaction) Sounds**	**Admission and discharge**	N/A	N/A	N/A	N/A	N/A	N/A	N/A	N/A
	**Activity of daily living: Non-mobilization**	12 min 1.00 %	4 min 1.00 %	8 min 2.00 %	0 min 0.0 %	25 min 2.08 %	19 min 4.75 %	5 min 1.25 %	1 min 0.25 %
	**Activity of daily living: Mobilization**	20 min 1.67 %	6 min 1.50 %	6 min 1.50 %	8 min 2.00 %	5 min 0.42 %	5 min 1.25 %	0 min 0.00 %	0 min 0.00 %
	**Nursing**	3 min 0.25 %	0 min 0.00 %	3 min 0.75 %	0 min 0.00 %	2 min 0.17 %	2 min 0.50 %	0 min 0.00 %	0 min 0.00 %
	**Diagnostic: Standard monitoring**	1 min 0.08 %	0 min 0.00 %	1 min 0.25 %	0 min 0.00 %	3 min 0.25 %	1 min 0.25 %	2 min 0.50 %	0 min 0.00 %

	**Diagnostic: Radiological diagnostic**	5 min 0.42 %	5 min 1.25 %	0 min 0.00 %	0 min 0.00 %	N/A	N/A	N/A	N/A
	**Diagnostic: Neurological interventions**	N/A	N/A	N/A	N/A	N/A	N/A	N/A	N/A
	**Ventilatory interventions: Intubation and extubation + Bronchoscopic interventions**	282 min 23.50 %	6 min 1.50 %	159 min 39.75 %	117 min 29.25 %	1 min 0.08 %	0 min 0.00 %	0 min 0.00 %	1 min 0.25 %
	**Cardiovascular interventions**	N/A	N/A	N/A	N/A	550 min 45.83 %	130 min 32.50 %	327 min 81.75 %	93 min 23.25 %
	**Renal intervention**	N/A	N/A	N/A	N/A	N/A	N/A	N/A	N/A
	**Unknown intervention**	2 min 0.17 %	0 min 0.00 %	2 min 0.50 %	0 min 0.00 %	N/A	N/A	N/A	N/A
	**Preparation board**	252 min 21.00 %	82 min 20.50 %	73 min 18.25 %	97 min 24.25 %	97 min 8.08 %	54 min 13.50 %	35 min 8.75 %	8 min 2.00 %
	**Free standing equipment**	91 min 7.58 %	19 min 4.75 %	40 min 10.00 %	32 min 8.00 %	58 min 4.83 %	41 min 10.25 %	6 min 1.50 %	11 min 2.75 %
	**Pendant**	221 min 18.42 %	79 min 19.75 %	72 min 18.00 %	70 min 17.50 %	168 min 14.00 %	69 min 17.25 %	52 min 13.00 %	47 min 11.75 %
	**Bed-related**	11 min 0.92 %	2 min 0.50 %	8 min 2.00 %	1 min 0.25 %	55 min 4.58 %	26 min 6.50 %	24 min 6.00 %	5 min 1.25 %
	**Privacy screens**	13 min 1.08 %	4 min 1.00 %	8 min 2.00 %	1 min 0.25 %	24 min 2.00 %	18 min 4.50 %	5 min 1.25 %	1 min 0.25 %
	**Short-lasting activities***	68 x	25 x	38 x	5 x	106 x	31 x	70 x	5 x
	**Continuous maintenance**	4 min 0.33 %	4 min 1.00 %	0 min 0.00 %	0 min 0.00 %	15 min 1.25 %	15 min 3.75 %	0 min 0.00 %	0 min 0.00 %
	**Short-lasting maintenance***	92 x	37 x	35 x	20 x	43 x	26 x	10 x	7 x
	**Clothing accessories**	128 min 10.67 %	44 min 11.00 %	46 min 11.50 %	38 min 9.50 %	66 min 5.50 %	38 min 9.50 %	13 min 3.25 %	15 min 3.75 %
	**Ringing***	3 x	0 x	3 x	0 x	20 x	7 x	13 x	0 x
**Observer**	**Observer talking**	N/A	N/A	N/A	N/A	9 min 0.75 %	8 min 2.00 %	1 min 0.25 %	0 min 0.00 %
	**Observer sound***	N/A	N/A	N/A	N/A	4 x	1 x	2 x	1 x

Supplementary Table 12. Sound source results for all categories, day 5. Friday, August 27th (07:00 a.m. - 07:00 a.m.). Total time was 24 h minus observer breaks, total = 20 h**.** Where there is no minute or percent value given, the number represents the number of occurrences. N/A indicates no occurrence of the category at that bed. Overall = 1200 min; Day, Evening, Night = 400 min.

	Description	Bed 1				Bed 2			
		Overall	Day	Evening	Night	Overall	Day	Evening	Night
Human (-Human) Sounds	Staff < 3 people talking (out of ward round)	509 min 42.42 %	201 min 50.25 %	129 min 32.25 %	179 min 44.75 %	140 min 11.67 %	65 min 16.25 %	6 min 1.50 %	69 min 17.25 %
	Staff ≥ 3 people talking (out of ward round)	117 min 9.75 %	59 min 14.75 %	40 min 10.00 %	18 min 4.50 %	30 min 2.50 %	14 min 3.50 %	0 min 0.00 %	16 min 4.00 %
	Staff during ward round < 3 people talking	7 min 0.58 %	0 min 0.00 %	7 min 1.75 %	0 min 0.00 %	2 min 0.17 %	2 min 0.50 %	0 min 0.00 %	0 min 0.00 %
	Staff during ward round ≥ 3 people talking	21 min 1.75 %	6 min 1.50 %	7 min 1.75 %	8 min 2.00 %	3 min 0.25 %	3 min 0.75 %	0 min 0.00 %	0 min 0.00 %
	Staff talking with patient	78 min 6.50 %	36 min 9.00 %	28 min 7.00 %	14 min 3.50 %	87 min 7.25 %	50 min 12.50 %	0 min 0.00 %	37 min 9.25 %
	Visitors, staff and patient talking < 3 people	N/A	N/A	N/A	N/A	3 min 0.25 %	3 min 0.75 %	0 min 0.00 %	0 min 0.00 %
	Visitors, staff and patient talking ≥ 3 people	30 min 2.50 %	0 min 0.00 %	30 min 7.50 %	0 min 0.00 %	N/A	N/A	N/A	N/A
	Staff sounds	2 min 0.17 %	0 min 0.00 %	2 min 0.50 %	0 min 0.00 %	1 min 0.08 %	1 min 0.25 %	0 min 0.00 %	0 min 0.00 %
	Patient sounds	2 min 0.17 %	2 min 0.50 %	0 min 0.00 %	0 min 0.00 %	8 min 0.67 %	7 min 1.75 %	0 min 0.00 %	1 min 0.25 %
	Visitor sounds	N/A	N/A	N/A	N/A	N/A	N/A	N/A	N/A
Object (-Human Interaction) Sounds	Admission and discharge	9 min 0.75 %	0 min 0.00 %	9 min 2.25 %	0 min 0.00 %	7 min 0.58 %	7 min 1.75 %	0 min 0.00 %	0 min 0.00 %
	Activity of daily living: Non-mobilization	22 min 1.83 %	22 min 5.50 %	0 min 0.00 %	0 min 0.00 %	12 min 1.00 %	12 min 3.00 %	0 min 0.00 %	0 min 0.00 %
	Activity of daily living: Mobilization	32 min 2.67 %	25 min 6.25 %	3 min 0.75 %	4 min 1.00 %	2 min 0.17 %	0 min 0.00 %	0 min 0.00 %	2 min 0.50 %
	Nursing	73 min 6.08 %	6 min 1.50 %	67 min 16.75 %	0 min 0.00 %	N/A	N/A	N/A	N/A

	**Diagnostic: Standard monitoring**	7 min 0.58 %	0 min 0.00 %	7 min 1.75 %	0 min 0.00 %	1 min 0.08 %	0 min 0.00 %	0 min 0.00 %	1 min 0.25 %
	**Diagnostic: Radiological diagnostic**	N/A	N/A	N/A	N/A	N/A	N/A	N/A	N/A
	**Diagnostic: Neurological interventions**	N/A	N/A	N/A	N/A	N/A	N/A	N/A	N/A
	**Ventilatory interventions: Intubation and extubation + Bronchoscopic interventions**	176 min 14.67 %	22 min 5.50 %	34 min 8.50 %	120 min 30.00 %	28 min 2.33 %	0 min 0.00 %	1 min 0.25 %	27 min 6.75 %
	**Cardiovascular interventions**	6 min 0.50 %	0 min 0.00 %	0 min 0.00 %	6 min 1.50 %	10 min 0.83 %	10 min 2.50 %	0 min 0.00 %	0 min 0.00 %
	**Renal intervention**	N/A	N/A	N/A	N/A	N/A	N/A	N/A	N/A
	**Unknown intervention**	6 min 0.50 %	2 min 0.50 %	4 min 1.00 %	0 min 0.00 %	N/A	N/A	N/A	N/A
	**Preparation board**	323 min 26.92 %	85 min 21.25 %	107 min 26.75 %	131 min 32.75 %	43 min 3.58 %	23 min 5.75 %	9 min 2.25 %	11 min 2.75 %
	**Free standing equipment**	104 min 8.67 %	21 min 5.25 %	52 min 13.00 %	31 min 7.75 %	61 min 5.08 %	14 min 3.50 %	3 min 0.75 %	44 min 11.0 %
	**Pendant**	274 min 22.83 %	100 min 25.00 %	106 min 26.50 %	68 min 17.00 %	95 min 7.92 %	39 min 9.75 %	5 min 1.25 %	51 min 12.75 %
	**Bed-related**	18 min 1.50 %	12 min 3.00 %	4 min 1.00 %	2 min 0.50 %	21 min 1.75 %	15 min 3.75 %	0 min 0.00 %	6 min 1.50 %
	**Privacy screens**	7 min 0.58 %	4 min 1.00 %	3 min 0.75 %	0 min 0.00 %	10 min 0.83 %	7 min 1.75 %	3 min 0.75 %	0 min 0.00 %
	**Short-lasting activities***	78 x	36 x	37 x	5 x	90 x	81 x	6 x	3 x
	**Continuous maintenance**	13 min 1.08 %	7 min 1.75 %	1 min 0.25 %	5 min 1.25 %	25 min 2.08 %	25 min 6.25 %	0 min 0.00 %	0 min 0.00 %
	**Short-lasting maintenance***	102 x	43 x	48 x	11 x	20 x	8 x	1 x	11 x
	**Clothing accessories**	187 min 15.58 %	31 min 7.75 %	127 min 31.75 %	29 min 7.25 %	35 min 2.92 %	12 min 3.00 %	11 min 2.75 %	12 min 3.00 %
	**Ringing***	23 x	8 x	9 x	6 x	12 x	5 x	0 x	7 x
**Observer**	**Observer talking**	N/A	N/A	N/A	N/A	13 min 1.08 %	6 min 1.50 %	7 min 1.75 %	0 min 0.00 %
	**Observer sound***	N/A	N/A	N/A	N/A	2 x	1 x	1 x	0 x

Supplementary Table 13. Sound source results for all categories, day 6. Saturday, August 28th (07:00 a.m. – 07:00 a.m.). Total time was 24 h minus observer breaks, total = 20 h. Where there is no minute or percent value given, the number represents the number of occurrences. N/A indicates no occurrence of the category at that bed. Overall = 1200 min; Day, Evening, Night = 400 min.

	Description	Bed 1				Bed 2			
		**Overall**	**Day**	**Evening**	**Night**	**Overall**	**Day**	**Evening**	**Night**
**Human (-Human) Sounds**	**Staff < 3 people talking (out of ward round)**	112 min 9.33 %	72 min 18.00 %	20 min 5.00 %	20 min 5.00 %	58 min 4.83 %	32 min 8.00 %	15 min 3.75 %	11 min 2.75 %
	**Staff ≥ 3 people talking (out of ward round)**	14 min 1.17 %	1 min 0.25 %	13 min 3.25 %	0 min 0.00 %	19 min 1.58 %	4 min 1.00 %	4 min 1.00 %	11 min 2.75 %
	**Staff during ward round < 3 people talking**	2 min 0.17 %	2 min 0.50 %	0 min 0.00 %	0 min 0.00 %	1 min 0.08 %	0 min 0.00 %	0 min 0.00 %	1 min 0.25 %
	**Staff during ward round ≥ 3 people talking**	N/A	N/A	N/A	N/A	1 min 0.08 %	1 min 0.25 %	0 min 0.00 %	0 min 0.00 %
	**Staff talking with patient**	3 min 0.25 %	3 min 0.75 %	0 min 0.00 %	0 min 0.00 %	144 min 12.00 %	116 min 29.00 %	19 min 4.75 %	9 min 2.25 %
	**Visitors, staff and patient talking < 3 people**	47 min 3.92 %	47 min 11.75 %	0 min 0.00 %	0 min 0.00 %	21 min 1.75 %	21 min 5.25 %	0 min 0.00 %	0 min 0.00 %
	**Visitors, staff and patient talking ≥ 3 people**	133 min 11.08 %	133 min 33.25 %	0 min 0.00 %	0 min 0.00 %	N/A	N/A	N/A	N/A
	**Staff sounds**	N/A	N/A	N/A	N/A	1 min 0.08 %	0 min 0.00 %	0 min 0.00 %	1 min 0.25 %
	**Patient sounds**	N/A	N/A	N/A	N/A	81 min 6.75 %	33 min 8.25 %	5 min 1.25 %	43 min 10.75 %
	**Visitor sounds**	6 min 0.50 %	6 min 1.50 %	0 min 0.00 %	0 min 0.00 %	N/A	N/A	N/A	N/A
**Object (-Human Interaction) Sounds**	**Admission and discharge**	N/A	N/A	N/A	N/A	19 min 1.58 %	0 min 0.00 %	6 min 1.50 %	13 min 3.25 %
	**Activity of daily living: Non-mobilization**	N/A	N/A	N/A	N/A	32 min 2.67 %	23 min 5.75 %	9 min 2.25 %	0 min 0.00 %
	**Activity of daily living: Mobilization**	N/A	N/A	N/A	N/A	9 min 0.75 %	9 min 2.25 %	0 min 0.00 %	0 min 0.00 %
	**Nursing**	39 min 3.25 %	39 min 9.75 %	0 min 0.00 %	0 min 0.00 %	5 min 0.42 %	5 min 1.25 %	0 min 0.00 %	0 min 0.00 %

	**Diagnostic: Standard monitoring**	N/A	N/A	N/A	N/A	12 min 1.00 %	10 min 2.50 %	2 min 0.50 %	0 min 0.00 %
	**Diagnostic: Radiological diagnostic**	N/A	N/A	N/A	N/A	3 min 0.25 %	3 min 0.75 %	0 min 0.00 %	0 min 0.00 %
	**Diagnostic: Neurological interventions**	N/A	N/A	N/A	N/A	N/A	N/A	N/A	N/A
	**Ventilatory interventions: Intubation and extubation + Bronchoscopic interventions**	1 min 0.08 %	0 min 0.00 %	1 min 0.25 %	0 min 0.00 %	1 min 0.08 %	0 min 0.00 %	0 min 0.00 %	1 min 0.25 %
	**Cardiovascular interventions**	N/A	N/A	N/A	N/A	N/A	N/A	N/A	N/A
	**Renal intervention**	N/A	N/A	N/A	N/A	N/A	N/A	N/A	N/A
	**Unknown intervention**	N/A	N/A	N/A	N/A	N/A	N/A	N/A	N/A
	**Preparation board**	179 min 14.92 %	70 min 17.50 %	54 min 13.50 %	55 min 13.75 %	38 min 3.17 %	31 min 7.75 %	0 min 0.00 %	7 min 1.75 %
	**Free standing equipment**	16 min 1.33 %	13 min 3.25 %	0 min 0.00 %	3 min 0.75 %	61 min 5.08 %	23 min 5.75 %	4 min 1.00 %	34 min 8.50 %
	**Pendant**	42 min 3.50 %	37 min 9.25 %	4 min 1.00 %	1 min 0.25 %	46 min 3.83 %	26 min 6.50 %	2 min 0.50 %	18 min 4.50 %
	**Bed-related**	7 min 0.58 %	7 min 1.75 %	0 min 0.00 %	0 min 0.00 %	13 min 1.08 %	11 min 2.75 %	2 min 0.50 %	0 min 0.00 %
	**Privacy screens**	14 min 1.17 %	12 min 3.00 %	1 min 0.25 %	1 min 0.25 %	9 min 0.75 %	5 min 1.25 %	4 min 1.00 %	0 min 0.00 %
	**Short-lasting activities***	97 x	94 x	3 x	0 x	10 x	9 x	1 x	0 x
	**Continuous maintenance**	25 min 2.08 %	7 min 1.75 %	17 min 4.25 %	1 min 0.25 %	19 min 1.58 %	4 min 1.00 %	15 min 3.75 %	0 min 0.00 %
	**Short-lasting maintenance***	45 x	38 x	6 x	1 x	31 x	25 x	0 x	6 x
	**Clothing accessories**	63 min 5.25 %	15 min 3.75 %	31 min 7.75 %	17 min 4.25 %	28 min 2.33 %	21 min 5.25 %	2 min 0.50 %	5 min 1.25 %
	**Ringing***	1 x	1 x	0 x	0 x	15 x	13 x	0 x	2 x
**Observer**	**Observer talking**	N/A	N/A	N/A	N/A	11 min 0.92 %	2 min 0.50 %	7 min 1.75 %	2 min 0.50 %
	**Observer sound***	N/A	N/A	N/A	N/A	2 x	0 x	2 x	0 x

Supplementary Table 14. Sound source results for all categories, day 7. Sunday, August 29th (07:00 a.m. - 07:00 a.m.). Total time was 24 h minus observer breaks, total = 20 h. Where there is no minute or percent value given, the number represents the number of occurrences. N/A indicates no occurrence of the category at that bed. Overall = 1200 min; Day, Evening, Night = 400 min.

	Description	Bed 1				Bed 2			
		Overall	Day	Evening	Night	Overall	Day	Evening	Night
Human (-Human) Sounds	Staff < 3 people talking (out of ward round)	264 min 22.00 %	27 min 6.75 %	91 min 22.75 %	146 min 36.50 %	279 min 23.25 %	170 min 42.50 %	100 min 25.00 %	9 min 2.25 %
	Staff ≥ 3 people talking (out of ward round)	23 min 1.92 %	0 min 0.00 %	7 min 1.75 %	16 min 4.00 %	94 min 7.83 %	64 min 16.00 %	30 min 7.50 %	0 min 0.00 %
	Staff during ward round < 3 people talking	N/A	N/A	N/A	N/A	6 min 0.50 %	4 min 1.00 %	2 min 0.50 %	0 min 0.00 %
	Staff during ward round ≥ 3 people talking	N/A	N/A	N/A	N/A	2 min 0.17 %	2 min 0.50 %	0 min 0.00 %	0 min 0.00 %
	Staff talking with patient	107 min 8.92 %	0 min 0.00 %	42 min 10.50 %	65 min 16.25 %	95 min 7.92 %	9 min 2.25 %	55 min 13.75 %	31 min 7.75 %
	Visitors, staff and patient talking < 3 people	N/A	N/A	N/A	N/A	3 min 0.25 %	0 min 0.00 %	3 min 0.75 %	0 min 0.00 %
	Visitors, staff and patient talking ≥ 3 people	N/A	N/A	N/A	N/A	N/A	N/A	N/A	N/A
	Staff sounds	5 min 0.42 %	0 min 0.00 %	5 min 1.25 %	0 min 0.00 %	12 min 1.00 %	6 min 1.50 %	5 min 1.25 %	1 min 0.25 %
	Patient sounds	51 min 4.25 %	0 min 0.00 %	5 min 1.25 %	46 min 11.50 %	142 min 11.83 %	10 min 2.50 %	52 min 13.00 %	80 min 20.00 %
	Visitor sounds	N/A	N/A	N/A	N/A	N/A	N/A	N/A	N/A
Object (-Human Interaction) Sounds	Admission and discharge	N/A	N/A	N/A	N/A	14 min 1.17 %	14 min 3.50 %	0 min 0.00 %	0 min 0.00 %
	Activity of daily living: Non-mobilization	8 min 0.67 %	0 min 0.00 %	3 min 0.75 %	5 min 1.25 %	N/A	N/A	N/A	N/A
	Activity of daily living: Mobilization	2 min 0.17 %	0 min 0.00 %	2 min 0.50 %	0 min 0.00 %	7 min 0.58 %	0 min 0.00 %	5 min 1.25 %	2 min 0.50 %
	Nursing	N/A	N/A	N/A	N/A	5 min 0.42 %	0 min 0.00 %	5 min 1.25 %	0 min 0.00 %

	**Diagnostic: Standard monitoring**	2 min 0.17 %	0 min 0.00 %	2 min 0.50 %	0 min 0.00 %	2 min 0.17 %	1 min 0.25 %	1 min 0.25 %	0 min 0.00 %
	**Diagnostic: Radiological diagnostic**	N/A	N/A	N/A	N/A	10 min 0.83 %	10 min 2.50 %	0 min 0.00 %	0 min 0.00 %
	**Diagnostic: Neurological interventions**	N/A	N/A	N/A	N/A	29 min 2.42 %	29 min 7.25 %	0 min 0.00 %	0 min 0.00 %
	**Ventilatory interventions: Intubation and extubation + Bronchoscopic interventions**	N/A	N/A	N/A	N/A	59 min 4.92 %	12 min 3.00 %	46 min 11.50 %	1 min 0.25 %
	**Cardiovascular interventions**	N/A	N/A	N/A	N/A	497 min 41.42 %	27 min 6.75 %	320 min 80.00 %	150 min 37.50 %
	**Renal intervention**	N/A	N/A	N/A	N/A	N/A	N/A	N/A	N/A
	**Unknown intervention**	N/A	N/A	N/A	N/A	N/A	N/A	N/A	N/A
	**Preparation board**	235 min 19.58 %	73 min 18.25 %	85 min 21.25 %	77 min 19.25 %	71 min 5.92 %	46 min 11.50 %	20 min 5.00 %	5 min 1.25 %
	**Free standing equipment**	88 min 7.33 %	14 min 3.50 %	17 min 4.25 %	57 min 14.25 %	110 min 9.17 %	45 min 11.25 %	27 min 6.75 %	38 min 9.50 %
	**Pendant**	73 min 6.08 %	1 min 0.25 %	48 min 12.00 %	24 min 6.00 %	194 min 16.17 %	101 min 25.25 %	73 min 18.25 %	20 min 5.00 %
	**Bed-related**	12 min 1.00 %	0 min 0.00 %	8 min 2.00 %	4 min 1.00 %	21 min 1.75 %	13 min 3.25 %	4 min 1.00 %	4 min 1.00 %
	**Privacy screens**	14 min 1.17 %	1 min 0.25 %	11 min 2.75 %	2 min 0.50 %	6 min 0.50 %	4 min 1.00 %	2 min 0.50 %	0 min 0.00 %
	**Short-lasting activities***	26 x	4 x	15 x	7 x	86 x	37 x	48 x	1 x
	**Continuous maintenance**	8 min 0.67 %	7 min 1.75 %	0 min 0.00 %	1 min 0.25 %	8 min 0.67 %	8 min 2.00 %	0 min 0.00 %	0 min 0.00 %
	**Short-lasting maintenance***	32 x	14 x	11 x	7 x	81 x	35 x	38 x	8 x
	**Clothing accessories**	116 min 9.67 %	44 min 11.00 %	50 min 12.50 %	22 min 5.50 %	127 min 10.58 %	45 min 11.25 %	75 min 18.75 %	7 min 1.75 %
	**Ringing***	9 x	1 x	6 x	2 x	44 x	21 x	23 x	0 x
**Observer**	**Observer talking**	N/A	N/A	N/A	N/A	8 min 0.67 %	1 min 0.25 %	7 min 1.75 %	0 min 0.00 %
	**Observer sound***	N/A	N/A	N/A	N/A	7 x	5 x	2 x	0 x

Supplementary Table 15. Sound source results for all categories, day 8. Monday, August 30th (07:00 a.m. - 09:15 a.m.). Total time was 2 h 15 min minus observer breaks, total =1 h 55 min. Where there is no minute or percent value given, the number represents the number of occurrences. N/A indicates no occurrence of the category at that bed. Overall = 115 min; *Day = 115 min (5h45min less than other days); Evening, Night = 0 min.

	Description	Bed 1				Bed 2			
		Overall	Day*	Evening	Night	Overall	Day*	Evening	Night
Human (-Human) Sounds	**Staff < 3 people talking (out of ward round)**	23 min 20.00 %	23 min 20.00 %			38 min 33.04 %	38 min 33.04 %		
	**Staff ≥ 3 people talking (out of ward round)**	3 min 2.61 %	3 min 2.61 %			1 min 0.87 %	1 min 0.87 %		
	**Staff during ward round < 3 people talking**	2 min 1.74 %	2 min 1.74 %			1 min 0.87 %	1 min 0.87 %		
	**Staff during ward round ≥ 3 people talking**	2 min 1.74 %	2 min 1.74 %			2 min 1.74 %	2 min 1.74 %		
	**Staff talking with patient**	9 min 7.83 %	9 min 7.83 %			7 min 6.09 %	7 min 6.09 %		
	**Visitors, staff and patient talking < 3 people**	N/A	N/A			N/A	N/A		
	**Visitors, staff and patient talking ≥ 3 people**	N/A	N/A			N/A	N/A		
	**Staff sounds**	N/A	N/A			1 min 0.87 %	1 min 0.87 %		
	**Patient sounds**	31 min 26.96 %	31 min 26.96 %			28 min 24.35 %	28 min 24.35 %		
	**Visitor sounds**	N/A	N/A			N/A	N/A		
Object (-Human Interaction) Sounds	**Admission and discharge**	N/A	N/A			N/A	N/A		
	**Activity of daily living: Non-mobilization**	1 min 0.87 %	1 min 0.87 %			N/A	N/A		
	**Activity of daily living: Mobilization**	N/A	N/A			N/A	N/A		
	**Nursing**	N/A	N/A			N/A	N/A		

	**Diagnostic: Standard monitoring**	N/A	N/A			1 min 0.87 %	1 min 0.87 %		
	**Diagnostic: Radiological diagnostic**	N/A	N/A			N/A	N/A		
	**Diagnostic: Neurological interventions**	N/A	N/A			N/A	N/A		
	**Ventilatory interventions: Intubation and extubation + Bronchoscopic interventions**	1 min 0.87 %	1 min 0.87 %			N/A	N/A		
	**Cardiovascular interventions**	N/A	N/A			101 min 87.83 %	101 min 87.83 %		
	**Renal intervention**	N/A	N/A			N/A	N/A		
	**Unknown intervention**	N/A	N/A			N/A	N/A		
	**Preparation board**	32 min 27.83 %	32 min 27.83 %			10 min 8.70 %	10 min 8.70 %		
	**Free standing equipment**	4 min 3.48 %	4 min 3.48 %			1 min 0.87 %	1 min 0.87 %		
	**Pendant**	9 min 7.83 %	9 min 7.83 %			25 min 21.74 %	25 min 21.74 %		
	**Bed-related**	1 min 0.87 %	1 min 0.87 %			1 min 0.87 %	1 min 0.87 %		
	**Privacy screens**	1 min 0.87 %	1 min 0.87 %			N/A	N/A		
	**Short-lasting activities***	3 x	3 x			3 x	3 x		
	**Continuous maintenance**	4 min 3.48 %	4 min 3.48 %			4 min 3.48 %	4 min 3.48 %		
	**Short-lasting maintenance***	2 x	2 x			7 x	7 x		
	**Clothing accessories**	12 min 10.43 %	12 min 10.43 %			6 min 5.22 %	6 min 5.22 %		
	**Ringing***	N/A	N/A			9 x	9 x		
**Observer**	**Observer talking**	N/A	N/A			4 min 3.48 %	4 min 3.48 %		
	**Observer sound***	N/A	N/A			N/A	N/A		
